# Sampling Strategies for Internal Validation Samples for Exposure Measurement–Error Correction: A Study of Visceral Adipose Tissue Measures Replaced by Waist Circumference Measures

**DOI:** 10.1093/aje/kwab114

**Published:** 2021-04-20

**Authors:** Linda Nab, Maarten van Smeden, Renée de Mutsert, Frits R Rosendaal, Rolf H H Groenwold

**Keywords:** internal validation sample, measurement-error correction, regression calibration, substitute exposure measurement

## Abstract

Statistical correction for measurement error in epidemiologic studies is possible, provided that information about the measurement error model and its parameters are available. Such information is commonly obtained from a randomly sampled internal validation sample. It is however unknown whether randomly sampling the internal validation sample is the optimal sampling strategy. We conducted a simulation study to investigate various internal validation sampling strategies in conjunction with regression calibration. Our simulation study showed that for an internal validation study sample of 40% of the main study’s sample size, stratified random and extremes sampling had a small efficiency gain over random sampling (10% and 12% decrease on average over all scenarios, respectively). The efficiency gain was more pronounced in smaller validation samples of 10% of the main study’s sample size (i.e., a 31% and 36% decrease on average over all scenarios, for stratified random and extremes sampling, respectively). To mitigate the bias due to measurement error in epidemiologic studies, small efficiency gains can be achieved for internal validation sampling strategies other than random, but only when measurement error is nondifferential. For regression calibration, the gain in efficiency is, however, at the cost of a higher percentage bias and lower coverage.

## Abbreviations

MCSE: Monte Carlo standard error
NEO: Netherlands Epidemiology of Obesity
VAT: visceral adipose tissue
WC: waist circumference

Preferred (or gold standard) measurements in large epidemiologic studies can be expensive, time consuming, invasive, or burdensome. These measures therefore are often replaced by simpler measures (less invasive, cheaper, faster), which are then assumed to correlate highly with the preferred measure. For example, consider studies of visceral adipose tissue (VAT) showing that higher values of VAT are associated with higher values of insulin resistance (1, 2). Measurement of VAT involves magnetic resonance imaging (MRI) scans. Alternatively, measurement of waist circumference (WC), which requires only a measuring tape, can provide a proxy measure of VAT (3). Nevertheless, the substitute measurements (e.g., WC) are not perfectly correlated with the gold standard (e.g., VAT) and, consequently, the substitute measurement can be viewed as an error-prone substitute for the gold standard.

Several methods have been developed to adjust for the bias in estimators of exposure-outcome associations when an exposure is measured with error (4–12). Despite the abundance of literature on methodology for measurement-error correction, its application is still rare (13, 14). Of the measurement error–correction methods in use, regression calibration is among the most common in epidemiologic research (15), possibly because of its relative simplicity and the ability to implement it in many situations (4, 7, 16, 17). Regression calibration relies on information about the relationship between the error-prone and the preferred (or gold standard) measurement (i.e., the measurement error model and its parameters). This relationship can be estimated using an internal validation sample, a subset of the main study including individuals for whom both the error-prone substitute and gold standard measurement are available.

Several regression calibration methods have been proposed. In linear models, examples include standard and validation regression calibration (see, for example, Carroll et al. (7)) as well as efficient regression calibration by Spiegelman et al. (18). The efficiency of these different regression calibration methods has been compared in simulation studies (for an example, see Thurston et al. (19)). Nonetheless, no studies have been conducted to investigate what internal-validation sampling strategy (e.g., random, stratified random, or extremes sampling) in conjunction with regression calibration provides the most efficient estimate of the corrected exposure-outcome association. The efficiency of regression calibration depends on the efficiency of the estimation of the calibration model, which might hypothetically be improved by sampling. for example, the extremes, assuming linear calibration models.

In the present study, we aimed to compare different sampling strategies for the internal validation sample in combination with different regression calibration methods to correct for the bias in exposure-outcome associations caused by measurement error. First, we introduce the Netherlands Epidemiology of Obesity (NEO) study and illustrate 3 different internal-validation sample sampling strategies. We then present a simulation study contrasting the finite sample properties of different sampling strategies of the internal validation sample in conjunction with regression calibration, motivated by the analysis of the NEO data. We conclude with a discussion of our results.

## CASE STUDY: VISCERAL ADIPOSE TISSUE MEASURES AS REPLACEMENT FOR WAIST CIRCUMFERENCE MEASURES

The NEO study is a large prospective observational cohort designed to investigate the pathways that lead to obesity-related diseases and conditions (20). Men and women aged 45–65 years with a self-reported body mass index of 27 or higher, living in the greater area of Leiden (in the West of the Netherlands), were eligible to participate in the NEO study. In addition, all inhabitants aged 45–65 years from one municipality (Leiderdorp) were invited, irrespective of their body mass index, to represent the general population.

**Figure 1:**
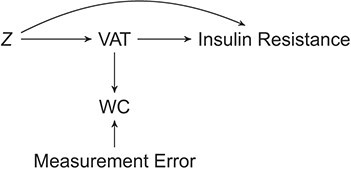
Assumptions of our motivating example. Error-prone waist circumference (WC) measures used as a substitute measure to estimate the association between visceral adipose tissue (VAT) and insulin resistance, confounded by *Z* (e.g., age, sex, total bodyfat).

A cross-sectional analysis of the association between VAT and insulin resistance was conducted in the subset of individuals that originated from the Leiderdorp subcohort of the NEO study. VAT depots were quantified by means of magnetic resonance imaging in a subsample of 668 (40%) individuals. These 668 individuals were randomly selected among the individuals who had no contraindication to undergo magnetic resonance imaging. WC was measured midway between the border of the lower costal margin and the iliac crest in all individuals. In this illustrative example we make 2 simplifying assumptions: 1) we consider WC measures as the error-prone substitute measure of the exposure of interest (i.e., VAT), and 2) we assume that WC is independent of insulin resistance given VAT and the confounding variables *Z* (i.e., nondifferential measurement error). These 2 assumptions are summarized in the causal diagram in Figure 1. Violation of the nondifferential measurement error assumption can lead to bias in both the regression calibration and internal validation analyses, under the circumstances explained in Results below. For the assessment of insulin resistance, the homeostatic model assessment of insulin resistance was used as fasting glucose (in mmol/L) × fasting insulin (in μ/L)/22.5. Of the 668 selected individuals, 19 were excluded from analysis because they used glucose-lowering therapy, and 1 additional patient was excluded because of a very low fasting blood glucose concentration. This resulted in including 648 individuals in our analysis. There were 22 missing values in the selected variables for analysis, which were imputed once (single imputation), using multivariate imputation through chained equations using the package mice, version 3.8.0 (21), with standard settings from the statistical software R (R Foundation for Statistical Computing, Vienna, Austria) (22). The association between VAT and insulin resistance was adjusted for the potential confounding variables age, sex, ethnicity, educational level, smoking status, alcohol consumption, total body fat, and physical activity, as well as for hormonal use and menopausal status in women. We refer to de Mutsert et al. (2) for further details on the assessment of all variables used in this study. Measures of VAT, WC, and total body fat were standardized, and measures of insulin resistance were log transformed. The effect sizes were derived from a linear regression analysis and expressed as percentage differences in outcome per standard deviationVAT.

After adjustment for confounding, insulin resistance was 27% higher (95% confidence interval: 19, 35) per standard deviation VAT (54 cm^2^). Alternatively, insulin resistance was 30% higher (95% confidence interval: 18, 43) per standard deviation WC (12 cm), with adjustment for the same potential confounders as the association between VAT and insulin resistance. Under the assumptions depicted in Figure 1, the difference in these 2 estimates can be explained by the measurement error in WC as a measure ofVAT.

### Testing sampling strategies in a resampling study

To illustrate sampling strategies for an internal validation sample in combination with regression calibration to correct for measurement error, a resampling study was performed using data of the 648 individuals from the Leiderdorp cohort among whom both VAT and WC measures were taken. Five hundred new data sets were created by sampling from the 648 individuals with replacement. In each of the 500 resampled data sets, the association between VAT and insulin resistance was estimated (referred to as the reference analysis). In addition, WC measurements were considered as a proxy for VAT and used to estimate the association between VAT and insulin resistance (referred to as the uncorrected analysis). Both analyses were adjusted for the same confounders as the original analysis presented above.

Next, 260 individuals (approximately 40% of 648) were included in the internal validation sample. This 40% was chosen to resemble the percentage of individuals for whom VAT depots were quantified of the whole Leiderdorp subcohort of the NEO study (i.e., in 668 individuals of the 1,670 individuals). The internal validation sample was sampled by using one of the following 3 sampling strategies: 1) random, 2) extremes, or 3) stratified random. The VAT measures of all individuals who were not selected in the internal validation sample were removed. In each of these data sets, the association between VAT and insulin resistance was estimated by using only the information from the 40% of individuals included in the internal validation sample (internal-validation-sample–restricted). Next, the VAT measurements available in the internal validation sample were used to correct for the measurement error in the association between WC and insulin resistance in 3 ways: 1) standard regression calibration, 2) validation regression calibration, or 3) efficient regression calibration.

For each sampling strategy and each regression calibration method, the mean of the 500 effect estimates was calculated and corresponding 95% confidence intervals were constructed based on the empirical standard errors. All analyses were adjusted for the above-mentioned potential confounders.

#### Sampling strategies and regression calibration methods.

**Figure 2:**
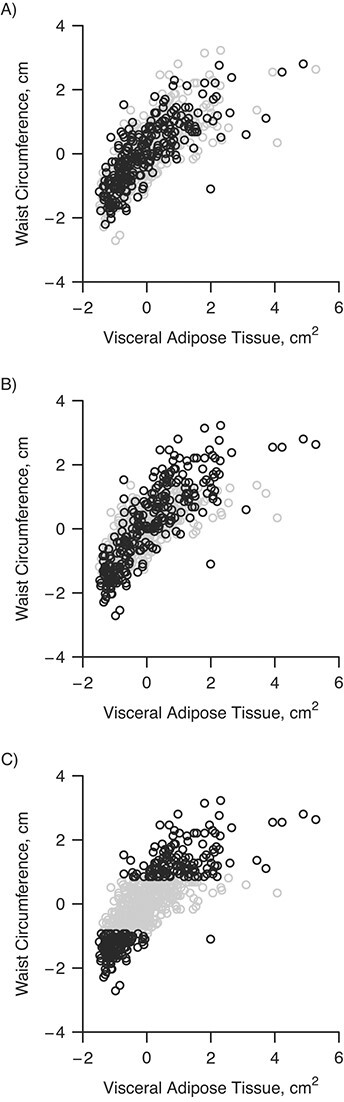
Visualization of different internal validation sample sampling strategies in the Leiderdorp cohort of the Netherlands Epidemiology of Obesity study, the Netherlands, 2008–2012. A) Visceral adipose tissue (VAT) measures are obtained at random (independent of waist circumference (WC)); B) VAT measures are obtained stratified randomly (stratified for strata of WC); and C) VAT measures are obtained in the individuals with the lowest and highest WC measures. The black points indicate the individuals included in the internal validation sample and the gray points the excluded individuals. The VAT measures and WC measures are standardized.

Figure 2 shows a visualization of the 3 sampling strategies used in this study. The internal validation sample was sampled: 1) randomly, 2) by grouping individuals according to tenths of the range of the measured WC values and sampling 26 individuals from each stratum (stratified random sampling), or 3) from the 130 individuals with the lowest and 130 with the highest measured WC values (extremes sampling). For stratified random sampling, when one of the strata contained less than 26 individuals, all individuals of this stratum were included in the internal validation sample. Subsequently, more than 26 individuals were sampled from the remaining strata, by equally distributing the shortage of individuals in the strata with fewer individuals among the strata with more individuals. We hypothesized that by sampling the extremes or by stratified random sampling, a linear relationship between WC and VAT could be estimated more efficiently in the internal validation set. By increasing the efficiency of the estimation of the linear relationship between WC and VAT, the efficiency of regression calibration was expected to increase simultaneously.

Three regression calibration methods were applied: 1) standard regression calibration, 2) validation regression calibration and 3) efficient regression calibration. Standard regression calibration and validation regression calibration are linear regressions where insulin resistance is regressed on a corrected version of the error-prone WC measures and the confounding variables. Standard regression calibration replaces the error-prone WC measures with the predicted mean of VAT given WC and the confounding variables. Validation regression calibration replaces the error-prone WC measures with the predicted mean of VAT given WC and confounding variables for individuals not included in the internal validation sample. For the individuals included in the internal validation sample, the error-prone WC measurements are replaced by their VAT measurements. Efficient regression calibration takes the inverse variance–weighted mean of the effect estimate of the internal-validation-sample–restricted analysis (see above) and the standard regression calibration analysis. Further technical details (including standard error estimation) can be found in the Web Appendix 1 (available at https://doi.org/10.1093/aje/kwab114).

**Table 1:** Estimated Association Between Visceral Adipose Tissue and Insulin Resistance^**a**^ in the Leiderdorp Cohort, Netherlands Epidemiology of Obesity Study, 2008–2012

	**Sampling Strategy**					
**Analytical Method**	**Random**		**Stratified Random**		**Extremes**	
	**Effect Size** ^**b**^ **, %**	**95% CI**	**Effect Size** ^**b**^ **, %**	**95% CI**	**Effect Size** ^**b**^ **, %**	**95% CI**
IVS restricted	26	14,40	20	9,33	18	7,31
Standard RC	67	24,126	60	25,105	59	24,104
Efficient RC	31	20,44	26	15,38	25	14,37
Validation RC	32	20,45	25	14,38	22	11,34

Abbreviations: CI, confidence interval; IVS, internal validation sample; RC, regression calibration.

^a^ Using different methods to correct for the measurement error when visceral adipose tissue measures were replaced by waist circumference measures.

^b^ Derived from β coefficients from linear regression analyses and expressed as percentage difference in outcome measure per standard deviation VAT; the effect size found in the reference analysis was 27% (95% CI: 19, 35), the effect size found in the uncorrected analysis was 30% (95% CI: 18, 43).

#### Results.

The results of the resampling study are shown in Table 1. In the uncorrected analysis, where WC was used to estimate the association between VAT and insulin resistance, the association between VAT and insulin resistance was overestimated compared with the reference analysis (30% vs. 27%). When the internal validation sample was sampled randomly, the internal-validation-sample–restricted analysis concurred with the reference analysis (26% vs. 27%). However, the standard regression calibration approach overestimated the association between VAT and insulin resistance severely in comparison with the reference analysis (67% vs. 27%). When the internal validation sample was sampled stratified randomly or by sampling the extremes, the internal-validation-sample–restricted analysis underestimated the association between VAT and insulin resistance in comparison with the reference analysis (20% and 18%, respectively, vs. 27%). In comparison, the standard regression calibration analysis, again, severely overestimated the association between VAT and insulin resistance (60% and 59% for stratified random and extremes sampling, respectively, vs. 27%). Further, our results suggest that stratified random and extremes sampling improve the estimates of efficient regression calibration and validation regression calibration, given that they appear to be closer to the reference analysis in comparison with random sampling, but this might be a chance finding due to cancelation of effects. Efficient and validation regression calibration are pooled averages of the underestimated association in the internal-validation-sample–restricted analysis and the overestimated association in the standard regression calibration analysis. Specifically, the results of the standard regression calibration analysis are clearly biased for all sampling strategies, and we therefore expect the results of the efficient and validation regression calibration analyses to be biased aswell.

**Figure 3:**
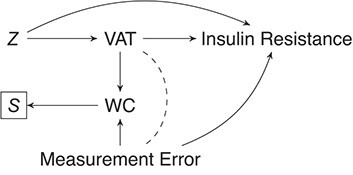
Collider stratification bias due to differential measurement error. Introduction of collider stratification bias when the data are observed (*S*) depending on the error-prone waist circumference (WC) measures with differential measurement error in a study estimating the association between (VAT) and insulin resistance, confounded by *Z* (e.g., age, sex, total bodyfat).

The results of our empirical example seem to indicate that only the internal-validation-sample–restricted analysis with a random sampling strategy concurs with the reference analysis. These results were not expected and can be explained by the fact that the measurement error in WC might depend on insulin resistance, given that WC measures also provide a proxy for subcutaneous fat, which in turn is associated with insulin resistance. Consequently, the assumption of nondifferential measurement error is violated. Particularly, to recover without bias the exposure-outcome association under study, regression calibration relies on the assumption that the measurement error is nondifferential. Furthermore, the internal-validation-sample–restricted analysis is biased when the internal validation sample is obtained by sampling stratified randomly or extremes. In this case, sampling stratified randomly or by the extremes introduced collider stratification bias, because inclusion in the internal validation sample is dependent on WC (depicted in the directed acyclic graph in Figure 3). Consequently, the relationship between VAT and insulin resistance is expected to be biased. Although sampling the internal validation sample other than randomly provides results that do not concur with the reference analysis here, general conclusions based on this empirical example are not warranted, which motivated our simulation study.

### Simulation study

A simulation study was conducted to evaluate the finite-sample properties of the different internal-validation-sample sampling strategies combined with regression calibration. The sample size and the values of the parameters of the data-generating mechanisms were similar to those estimated in the NEO subcohort described above.

#### Generating data.

Data sets were generated with a sample size of 650. The following data-generating mechanisms were used to generate data on sex, age, total body fat (TBF), VAT, WC, and insulin resistance (IR):

$$ {\displaystyle \begin{array}{l}\mathrm{sex}\sim \mathrm{Bern}\ (0.5),\kern1em \mathrm{age}\sim \mathrm{Unif}\ \left(45,65\right),\kern1em \\ \quad\mathrm{TBF}\mid \mathrm{sex},\mathrm{age}\sim N\left(-2+\mathrm{sex}+0.01\times \mathrm{age},0.5\right),\\{}\mathrm{VAT}=0.4-2\times \mathrm{sex}+0.01\times \mathrm{age}+0.9\\ \quad\times \ \mathrm{TBF}-\bigg(6\lambda \times\! \sqrt{\frac{0.5}{6\lambda}}\bigg)+\varepsilon, \ \ \varepsilon \sim \mathrm{Gamma} \left(6\lambda, \sqrt{\frac{0.5}{6\lambda}}\right)\!,\\{}\mathrm{WC}\mid \mathrm{VAT}\sim N\left(0.8\times \mathrm{VAT},{\tau}^2\right),\kern1em \mathrm{and}\\{}\mathrm{IR}\mid \mathrm{VAT},\mathrm{sex},\mathrm{age},\mathrm{TBF}\sim N\big(0.5+\beta \\ \quad\times \ \mathrm{VAT}-0.5\times \mathrm{sex}+0.01\times \mathrm{age}+0.3\times \mathrm{TBF},0.3\big).\end{array}} $$

The estimand of this simulation study is the conditional effect of VAT on insulin resistance (i.e., β) and was set to 0.2. The parameters $\tau$ and $\lambda$ were varied in different data-generation scenarios of our simulation study. The variance of the measurement error (i.e., $\tau$) was varied according to the explained variance of WC given VAT (hereafter referred to as *R*^2^). Values for *R*^2^ were set to 0.2, 0.4, 0.6, 0.8, and 0.9; corresponding values for $\tau$ can be found in Web Table 1a in Web Appendix 2. For reference, the *R*^2^ of the linear model of VAT and WC was approximately 0.6 in the NEO data. The above data-generating mechanism for VAT allowed changing the skewness of the residual errors while keeping the mean and variance of the marginal distribution constant. The skewness of the residual errors of VAT, $\varepsilon$ (hereafter referred to as skewness), was varied by changing $\lambda$. Values for the skewness were set to: 0.1, 1, 1.5, and 3; corresponding values for $\lambda$ can be found in Web Table 1b in Web Appendix 2. Additionally, we changed the distribution of $\mathrm{WC}\mid \mathrm{VAT}$ by using the square root of VAT instead of VAT to generate WC, in what was called the nonlinear scenario. *R*^2^, the skewness, and linearity were varied in a full-factorial design (i.e., $5\times 4\times 2=40$ scenarios). For each scenario, 5,000 data sets were generated.

#### Model estimation and performance measures.

In each generated data set, we applied the 3 sampling strategies (i.e., random, extremes, and stratified random sampling) and the 5 analyses (i.e., uncorrected, internal-validation-sample restricted, and the 3 regression calibration analyses). Standard errors were calculated using standard software or by using the multivariate delta method; see details in Web Appendix 1. Subsequently, Wald-based confidence intervals were constructed. Performance of the different analytical methods was evaluated in terms of bias, mean squared error (MSE), proportion of 95% confidence intervals that contain the true value of the estimand (coverage), empirical standard deviation of the estimated treatment effects, and square root of mean model-based variance of the estimated treatment effect. Monte Carlo standard errors (MCSE) were calculated for all performance measures (23), using the R package rsimsum, version 0.9.0 (24). All code used for the simulation study is publicly available (25).

#### Sensitivity analyses.

Two sensitivity analyses were conducted. First, to assess the sensitivity of our results to the size of the internal validation sample, we changed the percentage of individuals included to 10%, 25%, and 50%. Second, in our empirical example, it was seen that the performance of the 3 regression calibration analyses was generally poor. We hypothesized that this was possibly due to differential measurement error in the WC measures. Differential measurement error occurs when WC depends on the outcome insulin resistance, conditional on VAT and the confounding variables (see Web Appendix 1 for further details). To evaluate the impact of differential measurement error, a scenario was added by replacing the conditional distributions of WC and insulin resistance with:

$$ {\displaystyle \begin{array}{l}\mathrm{WC}\mid \mathrm{VAT}\sim N\left(\theta \times \mathrm{VAT}+\tau \times U,{\tau}^2\right)\kern1em \mathrm{and}\\[4pt] {}\mathrm{IR}\mid \mathrm{VAT},\mathrm{sex},\mathrm{age},\mathrm{TBF}\sim N\big(0.5+\beta \times \mathrm{VAT}-0.5\\[4pt] \quad\times \ \mathrm{sex}+0.01\times \mathrm{age}+0.3\times \mathrm{TBF}+\sqrt{0.3}\times U,0.3\big),\end{array}} $$

where *U* is a random variable with a Bernoulli distribution with mean 0.5. This scenario is an example of differential measurement error, because the distribution of the error-prone WC is dependent on the outcome insulin resistance via a third variable *U*, considered unmeasured. Here, $\tau$ was set equal to 0.44 (corresponding to an *R*^2^ of 0.8 in the main study), the skewness of the residual errors of VAT was 0.1, and the estimand ($\beta$) was again 0.2.

**Figure 4:**
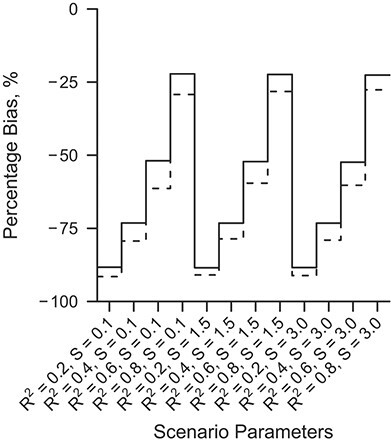
Nested loop plot of the percentage bias in the analysis ignoring measurement error, a simulation based on the Netherlands Epidemiology of Obesity study, the Netherlands, 2008–2012. Solid line, linear measurement error model; dashed line, nonlinear measurement error model. Order from outer to inner loops: Skewness of the residual errors of the gold standard measure (*S*, 3 levels, increasing); *R*^2^ of the measurement error model (4 levels, increasing).

### Results

For brevity, here we do not show results of the scenarios where *R*^2^ was equal to 0.9 or where skewness was equal to 1 (all results are presented in Web Figures 1–4 and Web Tables 2–9 in Web Appendix 3). The results of these parameter values did not contribute to the main comparisons made because the results of *R*^2^ equal to 0.9 were similar to *R*^2^ equal to 0.8 and the results of skewness equal to 1 were similar to skewness equal to 1.5. Further, because the focus of this work is the comparison between the 3 sampling strategies, we focus here on the performance of the 3 sampling strategies in the internal-validation-sample–restricted analysis and validation regression calibration. We chose to focus on validation regression calibration because this appears to be the standard method when applying regression calibration when there is an internal validation sample. The results of the sampling strategies using efficient regression calibration and standard regression calibration can be found in Web Appendix 3.

Figure 4 shows the percentage bias in the uncorrected analysis. In the uncorrected analysis, the association between VAT and insulin resistance was severely underestimated (bias ranging from −92% to −22%). The percentage bias decreased when *R*^2^ increased, and the bias in the uncorrected analysis was slightly higher when the measurement error model was nonlinear compared with a linear model. The skewness of the residual errors of VAT had no bearing onbias.

#### Efficiency in terms of mean squared errors.

**Figure 5:**
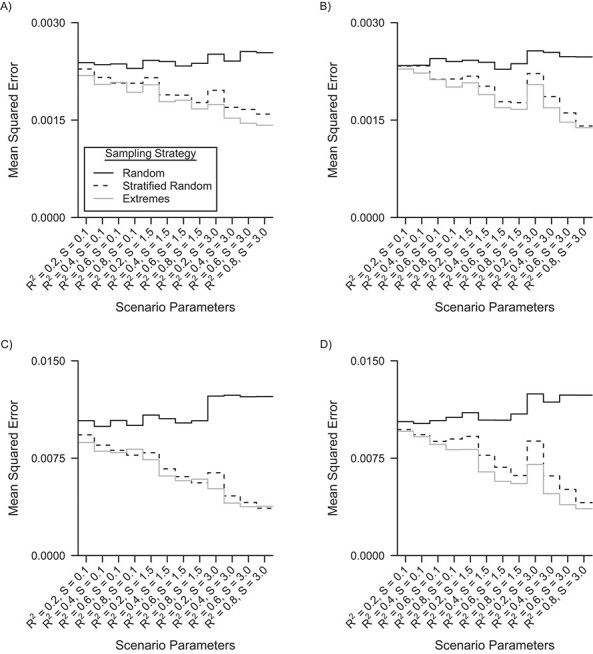
Nested loop plot of the mean squared errors in the analysis restricted to the internal validation sample for the 3 different sampling strategies, a simulation based on the Netherlands Epidemiology of Obesity study, the Netherlands, 2008–2012. A) Linear measurement error model and an internal validation sample of 40% of the main study; B) nonlinear measurement error model and an internal validation sample of 40% of the main study; C) linear measurement error model and an internal validation sample of 10% of the main study; and D) nonlinear measurement error model and an internal validation sample of 10% of the main study. Order from outer to inner loops: Skewness of the residual errors of the gold standard measure (*S*, 3 levels, increasing); *R*^2^ of the measurement error model (4 levels, increasing).

Figure 5 shows the mean squared errors for the internal validation sample restricted analysis with an internal validation sample of 40% and 10% of the main study’s sample size. Smaller mean squared errors were seen for stratified random and extremes sampling compared with random sampling for both samples sizes of the internal validation data. For the internal validation sample of 40% of the main study’s sample size, the percentage decrease in mean squared error was 19% and 24% on average, for stratified and extremes sampling, respectively; MCSE < 0.0001. For the internal validation sample of 10% of the main study’s sample size, the percentage decrease in mean squared error was 36% and 41% decrease on average, for stratified and extremes sampling, respectively; MCSE < 0.0005. Most notably, mean squared errors decreased further for both stratified random and extremes sampling when the residuals error of VAT were more skewed.

**Figure 6:**
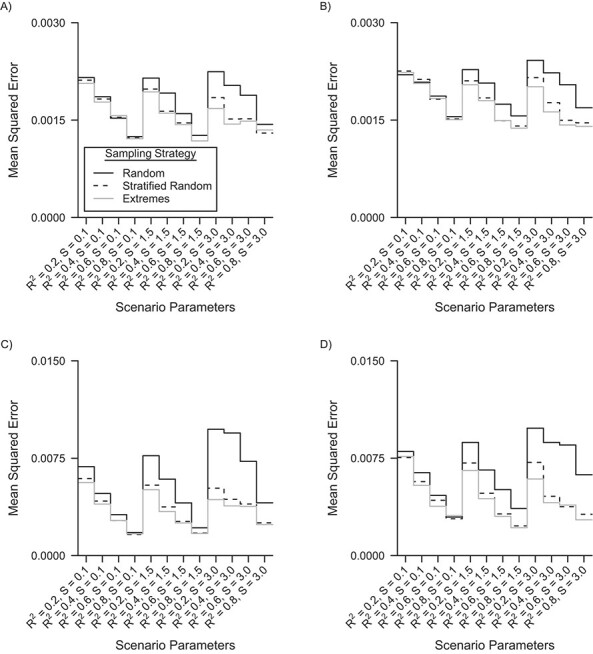
Nested loop plot of the mean squared errors in the analysis using validation regression calibration to correct for the measurement error for the 3 different sampling strategies, a simulation based on the Netherlands Epidemiology of Obesity study, the Netherlands, 2008–2012. A) Linear measurement error model and an internal validation sample of 40% of the main study; B) Nonlinear measurement error model and an internal validation sample of 40% of the main study; C) Linear measurement error model and an internal validation sample of 10% of the main study; and D) Nonlinear measurement error model and an internal validation sample of 10% of the main study. Order from outer to inner loops: Skewness of the residual errors of the gold standard measure (S, 3 levels, increasing); *R*^2^ of the measurement error model (4 levels, increasing).

Figure 6 shows the mean squared errors for validation regression calibration with an internal validation sample of 40% and 10% of the main study’s sample size. For the internal validation sample of 40% of the main study’s sample size, mean squared errors were smaller for stratified random and extremes sampling compared with random sampling, with a 10% and 12% decrease on average, respectively; MCSE < 0.0001. For the internal validation sample of 10% of the main study’s sample size, mean squared errors were found to be smaller for stratified random and extremes sampling compared with random sampling, with a 31% and 36% decrease on average, respectively; MCSE < 0.0005. The gain in efficiency was greatest for higher levels of skewness.

In a comparison between the internal validation restricted analysis and validation regression calibration, mean squared errors were generally smaller for validation regression calibration compared with the internal-validation-sample–restricted analysis (compare Figures 5 and 6). The difference was more pronounced for high values of the *R*^2^ and a validation sample of 10% of the main study’s samplesize.

#### Bias and coverage.

Table 2 shows percentage bias and coverage of the internal validation restricted and the validation regression calibration analysis with an internal validation sample of 40% of the main study’s sample size. For the internal-validation-sample–restricted analysis, all 3 different sampling strategies recovered the association between VAT and insulin resistance, with bias close to 0%. Additionally, coverage was close to the nominal level of 95% for all 3 sampling strategies. For the validation regression analysis and a randomly sampled internal validation sample, percentage bias was close to 0%. Contrary to random sampling, stratified random and extremes sampling introduced bias in the association under study, which was greater for higher levels of the skewness and the *R*^2^. Coverage was close to the nominal level of 95% for random sampling. For stratified random and extremes sampling, coverage was close to the nominal level of $95\%$ for all but the following 3 scenarios: There was undercoverage (stratified, 91.5% and 91.9%; extremes, 90.1% and 90.1%) in the linear setting when skewness was equal to 3.0 and *R*^2^ was 0.6 or 0.8, respectively. Additionally, there was undercoverage (stratified, 90%; extremes, 91.3%) in the nonlinear setting when the skewness was equal to 3.0 and *R*^2^ was 0.8.

Table 3 shows the percentage bias and coverage of the internal validation restricted and validation regression calibration analysis with an internal validation sample of 10% of the main study’s sample size. For the internal-validation-sample–restricted analysis and all 3 sampling strategies, percentage bias and coverage were both close to levels of 0% and 95%, respectively. For validation regression calibration, the association between VAT and insulin resistance was biased in most scenarios. Percentage bias in the association under study ranged between –5.0% and 7.2% when skewness was equal to 0.1. When skewness was equal to 1.5 or 3.0, percentage bias ranged between –24.5% and 10.2%. Since the association under study was biased in almost all scenarios, the effect estimate was undercovered for most scenarios, and increasingly when residual errors were more skewed, because bias was greater in these settings. For random sampling, the association under study was undercovered with levels ranging between 82.7% and 92.9%. For stratified random and extremes sampling, coverage was close to the nominal level of 95% when skewness was equal to 0.1 (ranging between 92.5% and 95.4%). When skewness was equal to 1.5 or 3.0, the effect estimate was generally undercovered with levels ranging between 62.9% and 94.6%.

**Table 2:** Percentage Bias and Coverage in the Estimated Association Between Visceral Adipose Tissue and Insulin Resistance With an Internal Validation Sample of 40% of the Main Study’s Sample Size^**a**^

			**Sampling Strategy**											
**Scenario**			**IVS-Restricted Analysis**						**Validation Regression Calibration**					
			**Percentage Bias** ^**b**^ **, %**			**Coverage** ^**c**^ **, %**			**Percentage Bias** ^**b**^ **, %**			**Coverage** ^**c**^ **, %**		
**Linear**	$\boldsymbol{{R}^2}$	**Skewness**	**R**	**SR**	**E**	**R**	**SR**	**E**	**R**	**SR**	**E**	**R**	**SR**	**E**
Yes	0.2	0.1	−0.5	0.2	−0.1	94.9	94.8	95.1	−0.5	0.2	−0.1	94.9	95.3	95.7
Yes	0.2	1.5	−0.1	−0.1	0.2	94.8	94.6	95.0	−0.4	−0.7	−0.3	94.8	95.5	95.6
Yes	0.2	3.0	−0.2	0.2	−0.1	94.7	94.4	94.7	−0.3	−1.2	−1.4	94.7	94.5	94.8
Yes	0.4	0.1	−0.1	0.4	0.1	95.0	95.3	94.9	−0.3	0.4	0.2	94.9	95.2	95.2
Yes	0.4	1.5	0.1	0.3	0.1	94.8	95.4	95.1	0.1	−1.7	−1.9	94.8	95.0	95.2
Yes	0.4	3.0	0.3	0.0	0.2	95.3	94.9	94.9	0.9	−4.1	−4.4	94.1	94.4	94.4
Yes	0.6	0.1	0.4	0.8	0.2	94.8	94.8	94.2	0.6	0.9	0.6	95.2	94.9	94.8
Yes	0.6	1.5	0.1	−0.3	0.4	95.1	95.0	94.5	0.5	−3.3	−3.3	94.7	94.5	94.4
Yes	0.6	3.0	0.0	−0.3	−0.1	94.8	94.8	94.6	0.9	−7.4	−8.9	93.2	91.5	90.8
Yes	0.8	0.1	−0.3	0.1	0.1	94.9	94.7	95.3	0.2	0.1	0.2	94.6	94.9	95.1
Yes	0.8	1.5	0.2	−0.2	−0.3	94.7	95.3	95.0	0.4	−3.6	−4.2	94.9	94.7	94.0
Yes	0.8	3.0	0.0	−0.2	0.0	94.7	94.7	94.7	1.0	−7.7	−9.5	93.8	91.9	90.8
No	0.2	0.1	0.3	0.2	0.2	94.8	94.6	95.1	−0.2	0.1	0.1	95.3	94.9	95.4
No	0.2	1.5	−0.3	0.2	−0.2	94.6	95.0	95.4	−0.7	−0.3	−0.5	94.7	95.2	95.5
No	0.2	3.0	−0.2	0.2	0.1	94.3	94.5	94.3	−0.5	−0.4	−0.4	94.7	94.7	94.7
No	0.4	0.1	0.4	0.0	−0.1	95.3	94.4	94.9	0.4	−0.1	−0.4	95.2	94.7	95.2
No	0.4	1.5	−0.6	−0.1	−0.2	94.8	95.4	95.0	−0.8	−1.3	−1.5	95.0	95.6	95.5
No	0.4	3.0	−0.2	−0.3	−0.4	94.6	94.3	94.4	−0.4	−2.7	−2.6	94.6	94.2	94.7
No	0.6	0.1	0.4	−0.4	−0.1	94.7	95.0	94.9	0.1	−0.5	−1.0	94.8	95.2	94.8
No	0.6	1.5	0.2	0.4	0.4	95.1	95.3	95.4	0.2	−2.2	−2.9	95.4	95.3	95.2
No	0.6	3.0	0.0	−0.1	0.0	94.6	94.8	94.5	0.2	−5.6	−5.6	94.0	93.5	93.5
No	0.8	0.1	0.1	0.0	0.3	94.5	94.8	94.8	0.4	0.1	0.3	94.4	94.8	95.2
No	0.8	1.5	0.0	−0.2	−0.2	94.9	94.4	94.8	−0.1	−5.7	−4.9	94.4	93.3	94.1
No	0.8	3.0	0.3	0.3	0.4	94.7	95.0	94.6	1.0	−9.7	−9.1	94.0	90.0	91.3

Abbreviations: E, extremes; IVS, internal validation sample; R, random; SR, stratified random.

^a^ Simulation based on the Netherlands Epidemiology of Obesity study, the Netherlands, 2008–2012.

^b^ Monte Carlo standard error < 0.001.

^c^ Monte Carlo standard error < 0.005.

**Table 3:** Percentage Bias and Coverage in the Estimated Association Between Visceral Adipose Tissue and Insulin Resistance With an Internal Validation Sample of 10% of the Main Study’s Sample Size^**a**^

			**Sampling Strategy**											
**Scenario**			**IVS-Restricted Analysis**						**Validation Regression Calibration**					
			**Percentage Bias** ^**b**^ **, %**			**Coverage** ^**c**^ **, %**			**Percentage Bias** ^**b**^ **, %**			**Coverage** ^**c**^ **, %**		
**Linear**	$\boldsymbol{{R}^2}$	**Skewness**	**R**	**SR**	**E**	**R**	**SR**	**E**	**R**	**SR**	**E**	**R**	**SR**	**E**
Yes	0.2	0.1	−0.9	0.3	−0.6	94.2	94.5	94.1	−1.4	0.1	−0.4	92.3	94.4	95.4
Yes	0.2	1.5	0.2	−0.4	0.0	94.2	95.0	94.0	−0.5	−4.0	−3.2	91.7	93.6	94.4
Yes	0.2	3.0	−0.2	0.2	0.2	94.5	94.5	94.6	1.4	−9.8	−8.1	89.1	89.5	91.3
Yes	0.4	0.1	0.1	0.5	1.0	94.8	94.4	94.7	1.7	1.6	1.3	91.7	93.3	93.4
Yes	0.4	1.5	−0.4	0.0	−0.3	95.1	94.8	94.4	3.9	−8.3	−8.2	89.7	89.3	91.5
Yes	0.4	3.0	−0.2	−0.2	−0.1	94.6	94.8	94.4	9.0	−20.1	−19.5	85.3	73.8	78.1
Yes	0.6	0.1	0.4	0.4	0.1	94.3	94.5	94.7	2.9	2.0	1.8	91.2	92.7	93.3
Yes	0.6	1.5	−0.1	−0.4	0.2	95.3	94.3	94.5	4.5	−10.9	−11.1	88.4	86.7	87.7
Yes	0.6	3.0	−0.2	−0.7	−0.2	94.3	94.0	94.4	10.2	−24.5	−24.5	82.7	62.9	65.5
Yes	0.8	0.1	0.0	−0.6	−0.3	94.9	94.7	94.6	1.0	0.4	0.8	92.9	93.7	93.6
Yes	0.8	1.5	−1.4	−0.5	−0.9	94.7	94.5	94.8	2.5	−9.8	−8.9	91.1	88.7	89.0
Yes	0.8	3.0	−0.2	0.2	0.3	94.3	94.7	94.7	7.6	−19.0	−18.1	85.5	73.7	76.5
No	0.2	0.1	0.3	−0.7	1.1	94.3	94.0	94.3	−5.0	−1.7	−0.5	92.9	94.2	94.9
No	0.2	1.5	−0.1	0.1	−0.2	94.7	94.6	94.4	−3.7	−2.5	−3.0	92.2	94.2	94.6
No	0.2	3.0	−1.0	1.3	−0.2	94.2	94.0	94.5	−3.7	−2.5	−3.8	91.9	93.5	94.2
No	0.4	0.1	0.6	0.0	0.4	94.8	94.5	94.0	0.6	0.7	−1.7	92.3	93.9	93.9
No	0.4	1.5	−1.5	0.5	−1.0	94.3	94.5	94.5	−0.4	−4.0	−8.7	91.4	93.0	92.7
No	0.4	3.0	−0.4	−0.1	−0.1	94.9	94.4	95.0	2.8	−10.2	−14.2	89.6	89.1	87.8
No	0.6	0.1	0.6	0.0	−0.1	94.7	94.7	94.2	1.2	2.3	−1.6	91.5	93.4	93.5
No	0.6	1.5	0.2	0.1	0.3	94.9	94.7	94.9	3.5	−6.0	−10.8	90.5	92.0	91.2
No	0.6	3.0	−0.2	0.8	0.0	94.0	94.5	94.0	7.7	−16.4	−21.8	85.5	80.3	75.9
No	0.8	0.1	−0.3	−0.2	0.4	93.7	94.2	94.2	2.0	4.1	7.2	91.6	92.6	92.5
No	0.8	1.5	−0.8	−0.3	−0.5	94.3	94.0	94.2	3.2	−8.6	−6.6	88.4	89.1	91.6
No	0.8	3.0	0.3	0.4	0.7	94.6	94.9	94.4	8.8	−20.2	−18.3	83.5	71.2	77.7

Abbreviations: E, extremes; IVS, internal validation sample; R, random; SR, stratified random.

^a^ Simulation based on the Netherlands Epidemiology of Obesity study, the Netherlands, 2008–2012.

^b^ Monte Carlo standard error < 0.005.

^c^ Monte Carlo standard error < 0.01.

The results for the internal-validation-sample–restricted analysis and validation regression calibration with an internal validation sample composed of 25% of the main study can be found in Web Appendix 3.

#### Differential measurement error.

Table 4 shows that differential measurement error can cause bias in the association between VAT and insulin resistance. The internal-validation-sample–restricted analysis using internal validation data that is sampled randomly recovered the association under study with percentage bias equal to 0%. The internal-validation-sample–restricted analysis using stratified random or extremes sampling was biased in both cases, with percentage bias equal to 10% and 30%, respectively. The different regression calibration analyses were all biased, independent of how the internal validation sample was sampled.

**Table 4:** Percentage Bias According to Sampling Strategy in the Estimated Association Between Visceral Adipose Tissue and Insulin Resistance in Case of Differential Measurement Error^**a**^

	**Percentage Bias According to Sampling Strategy** ^**b**^ **, %**		
**Analytical Method**	**Random**	**Stratified Random**	**Extremes**
IVS restricted	0	10	30
Standard RC	76	75	75
Efficient RC	42	45	46
Validation RC	35	36	36

Abbreviations: IVS, internal validation sample; RC, regression calibration.

^a^ Simulation based on the Netherlands Epidemiology of Obesity study, the Netherlands, 2008–2012.

^b^ The percentage bias in the uncorrected analysis was 25%, Monte Carlo standard error < 0.001 for all analyses.

## DISCUSSION

This study investigated 3 internal validation sampling strategies (random, stratified random, and extremes sampling) in conjunction with regression calibration to correct for measurement error in a continuous covariate. Our simulation study showed a small efficiency gain in terms of mean squared error of stratified random and extremes sampling over a random sampling strategy for the internal-validation-sample–restricted and regression calibration analyses but only when measurement error was nondifferential. For regression calibration, this gain in efficiency was at the cost of higher percentage bias and lower confidence interval coverage. We therefore recommend that, in general, regression calibration using randomly sampled validation samples are preferable over stratified or extremes sampled samples.

Three different regression calibration methods (i.e., standard, efficient, and validation) and an internal-validation-sample–restricted analysis were tested in our simulation study. The internal-validation-sample–restricted analysis and validation regression calibration showed the best overall performance in terms of percentage bias and confidence interval coverage of the true effect. Furthermore, validation regression calibration had the same efficiency as efficient regression calibration under strong correlations between the exposure and outcome. These findings are consistent with the work by Thurston et al. (19). What is more, a slight undercoverage of the confidence intervals was found for the efficient regression calibration approach.

In addition, our simulation study showed a gain in efficiency of validation regression calibration over the internal-validation-sample–restricted analysis. The gain in efficiency is more pronounced when the *R*^2^ of the measurement error model is high and for smaller validation samples (e.g., 10% of full sample). Intuitively, the validation sample–restricted analysis uses information about the gold standard measurement but only for those individuals in whom it was measured (i.e., the internal validation sample). For regression calibration, however, information about all individuals is used, which tends to increase the efficiency, compared with the restricted analysis. However, the efficiency is negatively affected by the uncertainty in the correction factor that needs to be estimated from the internal validation sample. The relative gain in efficiency for regression calibration compared with an analysis of the gold standard measurement only (restricted to the validation sample) depends on the correlation between the gold standard and the error-prone measurement (15), as well as the appropriateness of parametric assumptions made for regression calibration.

Related work on internal validation studies can be found in the field of psychology, often referred to there as “2-method designs” or “planned missing data designs.” These terms were recently suggested by Rioux et al. (26) for epidemiologic research. Graham et al. (27) studied the cost-effectiveness of 2-method designs and concluded that, in comparison with an analysis restricted to the internal validation sample, the 2-method design can yield lower standard errors for testing associations using structural equation modeling. In particular, the benefit of the design can be enormous when there is a large cost difference between the error-prone and the gold standard measures and effect sizes are small.

Regression calibration is one approach to correct for measurement error. Other methods for measurement-error correction include multiple imputation for measurement error (8), simulation-extrapolation (9), Bayesian methods (5), and methods based on maximum likelihood estimation (28). Earlier simulation studies have been conducted comparing multiple imputation for measurement error and regression calibration. These studies showed that, in general, multiple imputation for measurement error produced less-biased estimates than regression calibration but can have larger variances (8, 29, 30). Simulation-extrapolation was originally designed to correct for measurement error that is random, which the measurement error in our case study was not. Adaptations have been made to also allow for systematic measurement error (31).

In our motivating example, regression calibration performed poorly. This was likely caused by violation of the nondifferential measurement error assumption that regression calibration relies on and it signifies the importance of this assumption. WC measures might contain differential measurement error, because WC measures also provide a proxy for subcutaneous fat, which in turn is associated with insulin resistance. In our simulation study, where measurement error was known to be nondifferential or differential, regression calibration performed well (nondifferential measurement error) or poorly (for differential measurement error), which further adds to our suspicion that differential measurement error might have affected the results of the motivating example.

Nondifferential measurement error is a strong assumption and might be unlikely in practice (32). Our motivating example signifies the importance of this assumption for measurement-error correction and illustrates that when measurement error is differential, 1) regression calibration is not an appropriate method for measurement-error correction, and 2) nonrandom internal validation sampling strategies introduce collider stratification bias (see Figure 3). In the situation where differential measurement error is assumed, alternative methods for measurement-error correction can be used—for example, multiple imputation for measurement error (8) and regular multiple imputation methods (33–35). Future research could investigate whether nonrandom validation sample strategies improve the efficiency of multiple imputation methods for measurement-error correction.

Large epidemiologic studies could consider using internal validation samples when a gold standard measurement is expensive, time consuming, or burdensome. Our publicly available code (25), provides an opportunity for careful planning of a sampling strategy, including the size of the internal validation sample, and the choice between an analysis restricted to the internal validation sample or application of regression calibration. The code can be adapted to accommodate situations other than those studiedhere.

In summary, our study showed that there appears to be little added value to stratified random or extremes sampling in internal validation studies to correct for measurement error. Regression calibration, if nondifferential measurement error can be assumed, was shown to be an effective approach to correct analyses for measurement error. When handled with care, application of regression calibration can improve efficiency of epidemiologic studies with internal validation samples.

## Supplementary Material

Web_Material_kwab114
