# Supplementary material for: Sampling Strategies for Internal Validation Samples for Exposure Measurement–Error Correction: A Study of Visceral Adipose Tissue Measures Replaced by Waist Circumference Measures
Source: Am J Epidemiol. 2021 Apr 20;190(9):1935–47. doi: 10.1093/aje/kwab114 (PMC8408354; doi:10.1093/aje/kwab114)
Supplement: Web_Material_kwab114 [file web_material_kwab114.pdf]

Web Material

Sampling Strategies for Internal Validation Samples for Exposure Measurement Error Correction: a Study of Visceral Adipose Tissue Measures Replaced by Waist Circumference Measures

Linda Nab, Maarten van Smeden, Renée de Mutsert, Frits R. Rosendaal, and Rolf H.H. Groenwold

Contents

1 Web Appendix 1 1
2 Web Appendix 2 2
3 Web Appendix 3 3

List of Figures

1 Web Figure 1 4
2 Web Figure 2 6
3 Web Figure 3 10
4 Web Figure 4 11

List of Tables

1 Web Table 1 3
2 Web Table 2 4
3 Web Table 3 5
4 Web Table 4 7
5 Web Table 5 8
6 Web Table 6 9
7 Web Table 7 12
8 Web Table 8 13
9 Web Table 9 14

Web Appendix 1

Throughout the main paper, our interest is the causal effect of the exposure VAT on the outcome insulin resistance IR, adjusted for a predefined set of k confounders, jointly written as $\boldsymbol{Z}$ (e.g., age, sex and total body fat). We assume a linear model for the outcome without interaction between exposure and covariates:

$$\text{IR} = \text{intercept} + \beta\text{VAT} + \boldsymbol{\gamma}^{'}\boldsymbol{Z} + \varepsilon. \tag{W1}$$

Here, we assume that the residuals errors ε are independent of VAT and confounders $\boldsymbol{Z}$, with mean 0 and variance σ^2. Additionally, $\boldsymbol{\gamma}$ is assumed a $k \times 1$ vector of regression coefficients. The parameter β in equation (W1) is the parameter of interest. We consider the setting that instead of the exposure of interest, VAT, WC is measured. The variable WC is the error-prone substitute measure for VAT, where we assume that WC $= \theta_1$VAT $+ U$, where U is a random variable, with mean 0 and variance τ^2, and U is assumed independent of VAT. The factor θ_1 is a scalar, used to scale VAT to the same scale as WC. We also assume *non-differential* measurement error, i.e., $\text{WC} \perp\!\!\!\perp Y|\text{VAT}, \boldsymbol{Z}$. This form of measurement error is referred to as random (or sometimes classical) measurement error if $\theta = 1$ and systematic (or sometimes linear) measurement error otherwise [1, 2]. Since the substitute measure is often measured on a different scale than the true measure, measurement error will often be of the systematic form. Using WC instead of VAT in the linear model yields:

$$\text{E}[\text{IR}|\text{WC}, \boldsymbol{Z}] = \text{intercept}^* + \beta^*\text{WC} + \boldsymbol{\gamma}^{*'}\boldsymbol{Z}. \tag{W2}$$

Under this model, by the law of total expectation, we have $\mathbb{E}[\text{IR}|\text{WC}, \boldsymbol{Z}] = \text{intercept} + \beta \times \mathbb{E}[\text{VAT}|\text{WC}, \boldsymbol{Z}] + \boldsymbol{\gamma}^*\boldsymbol{Z}$, which relies on the assumption that the measurement error is non-differential [3]. It follows that,

$$\beta^* = \alpha\beta \quad \text{with} \quad \alpha = \frac{\mathbb{C}\text{ov}(\text{WC}, \text{VAT}|\boldsymbol{Z})}{\mathbb{V}\text{ar}(\text{WC}|\boldsymbol{Z})} = \frac{\theta_1\, \mathbb{V}\text{ar}(\text{VAT}|\boldsymbol{Z})}{\theta_1^2\, \mathbb{V}\text{ar}(\text{VAT}|\boldsymbol{Z}) + U}. \tag{W3}$$

In conclusion, the ordinary least squared estimator for β^* is biased for β by a factor α. This factor is sometimes referred to as the attenuation factor in case of random measurement error, since $\mathbb{V}\text{ar}(\text{VAT}|\boldsymbol{Z}) < \mathbb{V}\text{ar}(\text{VAT}|\boldsymbol{Z}) + U$ and hence, $\alpha < 1$.

The different analyses with internal validation samples

When a study contains an internal validation sample for which information is available on both WC and VAT, different analyses can be conducted. Five different estimators are explained below. The variance of these estimators can be obtained from standard output of statistical software when no further details on variance estimation are provided below. The internal validation sample restricted analysis relies on the assumption that the VAT measures in the main study are completely missing at random and the regression calibration methods rely on the assumption that measurement error in WC is non-differential.

Uncorrected analysis. The measurement error is ignored and the relation between VAT and IR is estimated using the error-prone substitute measure WC. Under the assumptions above, as shown in equation W3, this estimator is biased by a factor α.

Internal validation sample restricted analysis. The association between VAT and IR is determined using only the data from the internal validation sample (in which a direct measure of VAT is available). This approach will naturally yield unbiased estimates if measures of VAT are missing completely at random in the main study, but power of the study will substantially decrease as only a part of the data available in the main study is used.

Standard regression calibration. The basis of regression calibration is the replacement of WC by a corrected version of WC, based on the regression of VAT on WC and the confounders $\boldsymbol{Z}$. In this way, the induced measurement error in the uncorrected analysis is corrected by regressing the outcome IR on the confounders $\boldsymbol{Z}$ and $\mathbb{E}[\text{VAT}|\text{WC}, \boldsymbol{Z}]$ instead of WC (i.e., by using the predicted values from regressing VAT on WC and $\boldsymbol{Z}$, instead of WC). This method is identical to dividing the least squares estimator β^* in equation W2 by the correction factor α defined in equation W3 [2]. The variance of this estimator can be estimated by applying the Delta method described by Rosner et al. [4].

Efficient regression calibration. This analysis pools the estimator of the internal validation sample restricted analysis with the regression calibration estimator, by using weights equal to the inverse of the variance of the two estimates, and was described by Spiegelman et al. [5]. This approach is called efficient regression calibration since it makes use of the fact that in the individuals included in the internal validation sample, VAT is actually known and does not neglect this information. The variance of this estimator can be estimated by taking the inverse of: the sum of the inverse of the variance of the internal validation sample restricted estimator and the inverse of the variance of the regression calibration estimator, as described by Spiegelman et al. [5].

Validation regression calibration. This analysis uses the predicted values from regressing VAT on WC and $\boldsymbol{Z}$ for individuals outside the internal validation sample and VAT otherwise. We call this approach validation regression calibration approach since this is the standard regression calibration approach in internal validation studies [1]. Validation regression calibration treats the predicted values as if they were known and therefore neglects their uncertainty.

Web Appendix 2

In the simulation study presented in the main text, the measurement error variance τ and the parameter λ in the gamma distribution of the residual errors of VAT were varied according to the R-squared of the measurement error model and skewness of the residuals errors, respectively. The corresponding values for τ and λ in the data generating mechanism found in the main text can be found in Web Table 1.

Web Table 1. Values of the parameters R-squared and skewness varied in the simulation study in a full factorial design. The values for τ and λ present the values for that parameter in the data generating mechanism that corresponds to the given R-squared and skewness, respectively.

(a) R-squared and corresponding τ

R-squared	τ
0.2	1.8
0.4	1.1
0.6	0.7
0.8	0.4
0.9	0.3

(b) Skewness and corresponding λ

Skewness	λ
0.1	65.6
1	0.7
1.5	0.3
3	0.1

Web Appendix 3

The results of the simulation study that were left out the main text for brevity are shown in the following subsections. Full results of the simulation study can also be found at an online repository [6]. Specifically, `R` compatible summary files are available at `www.github.com/LindaNab/me_neo/results/summaries`. These summary files contain more detailed information on e.g. model based standard errors, empirical standard errors and Monte Carlo standard errors. Additionally, output of each single run of the simulation study can be found at `www.github.com/LindaNab/me_neo/data/output` and subsequent folders.

Internal validation restricted analysis

The main results of the internal validation restricted analysis were shown in the main text. Web Figure 1 shows the mean squared error of the association between visceral adipose tissue and insulin resistance using an internal validation sample of 25% of the main study's sample size. Web Table 2 shows the mean squared error of the association under study in the scenarios where R-squared was equal to 0.9 or skewness was equal to 1.0, that were left out the main text for brevity. Web Table 3 shows the percentage bias and coverage of the association under study in the scenarios where R-squared was equal to 0.9 or skewness was equal to 1.0.

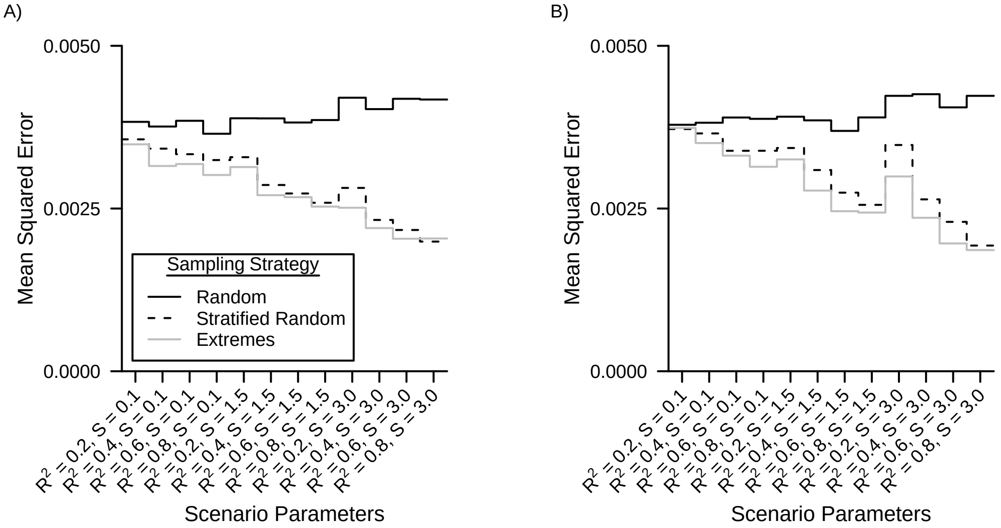

Web Figure 1. Nested loop plot of the mean squared errors in the analysis restricted to the internal validation sample for the three different sampling strategies. A) Linear measurement error model and an internal validation sample of 25% of the main study; and B) Non-linear measurement error model and an internal validation sample of 25% of the main study. Order from outer to inner loops: Skewness of the residual errors of the gold standard measure (S, 3 levels, increasing); R-squared of the measurement error model (R^2, 4 levels, increasing).

Web Table 2. Mean squared error of the estimated association between visceral adipose tissue and insulin resistance in the analysis restricted to the internal validation sample

	Scenario		IVS[a] 40% of main study			IVS[a] 25% of main study			IVS[a] 10% of main study		
**Linear**	**Skewness**	R^2	Mean squared error[b]			Mean squared error[b]			Mean squared error[b]		
			R	*SR*	*E*	*R*	*SR*	*E*	*R*	*SR*	*E*
**yes**	**0.1**	**0.9**	0.0023	0.0020	0.0019	0.0039	0.0031	0.0031	0.0100	0.0080	0.0084
**yes**	**1**	**0.2**	0.0024	0.0022	0.0022	0.0038	0.0034	0.0033	0.0104	0.0086	0.0080
**yes**	**1**	**0.4**	0.0024	0.0021	0.0020	0.0038	0.0031	0.0031	0.0103	0.0077	0.0069
**yes**	**1**	**0.6**	0.0024	0.0019	0.0018	0.0039	0.0029	0.0027	0.0100	0.0068	0.0067
**yes**	**1**	**0.8**	0.0023	0.0019	0.0018	0.0038	0.0029	0.0027	0.0106	0.0066	0.0070
**yes**	**1**	**0.9**	0.0024	0.0019	0.0018	0.0039	0.0028	0.0029	0.0103	0.0064	0.0071
**yes**	**1.5**	**0.9**	0.0024	0.0018	0.0017	0.0039	0.0025	0.0026	0.0109	0.0052	0.0059
**yes**	**3**	**0.9**	0.0024	0.0015	0.0013	0.0040	0.0020	0.0019	0.0120	0.0036	0.0040
**no**	**0.1**	**0.9**	0.0023	0.0020	0.0019	0.0038	0.0032	0.0031	0.0100	0.0089	0.0081
**no**	**1**	**0.2**	0.0024	0.0022	0.0022	0.0040	0.0036	0.0034	0.0104	0.0094	0.0085
**no**	**1**	**0.4**	0.0024	0.0021	0.0020	0.0038	0.0033	0.0031	0.0107	0.0084	0.0074
**no**	**1**	**0.6**	0.0024	0.0019	0.0019	0.0038	0.0029	0.0028	0.0102	0.0074	0.0068
**no**	**1**	**0.8**	0.0024	0.0019	0.0018	0.0039	0.0029	0.0027	0.0103	0.0068	0.0064
**no**	**1**	**0.9**	0.0023	0.0018	0.0017	0.0039	0.0027	0.0026	0.0103	0.0069	0.0064
**no**	**1.5**	**0.9**	0.0024	0.0016	0.0016	0.0039	0.0024	0.0023	0.0108	0.0060	0.0053
**no**	**3**	**0.9**	0.0026	0.0015	0.0014	0.0042	0.0019	0.0019	0.0123	0.0038	0.0036

[a] Internal validation sample
[b] For varying sampling strategies of the internal validation sample, R: random, SR: stratified random, E: extremes

Web Table 3. Percentage bias and coverage of the estimated association between visceral adipose tissue and insulin resistance in the analysis restricted to the internal validation sample

**Scenario**			**IVS[a] 40% of main study**						**IVS[a] 25% of main study**						**IVS[a] 10% of main study**					
**Linear**	**Skewness**	R^2	Percentage bias[b] (%)			Coverage[b] (%)			Percentage bias[b] (%)			Coverage[b] (%)			Percentage bias[b] (%)			Coverage[b] (%)		
			R	*SR*	*E*	*R*	*SR*	*E*	*R*	*SR*	*E*	*R*	*SR*	*E*	*R*	*SR*	*E*	*R*	*SR*	*E*
yes	0.1	0.9	-0.3	0.0	0.5	95.1	94.9	95.1	-0.5	-0.3	0.5	94.9	94.8	94.9	-0.7	-0.3	0.1	94.6	93.8	94.7
yes	1	0.2	0.3	-0.1	0.1	94.9	94.6	94.8	0.1	-0.4	0.0	95.2	94.2	94.2	0.9	-0.9	0.2	94.5	94.7	94.3
yes	1	0.4	-0.1	-0.6	-0.3	94.7	94.9	94.5	0.2	-0.7	-0.7	94.7	94.9	94.1	-0.6	-0.4	-0.3	94.7	94.5	94.3
yes	1	0.6	0.0	-0.2	-0.4	95.1	94.6	95.1	-0.2	-0.6	-0.2	94.6	94.4	95.3	0.8	-0.3	-0.3	94.8	94.7	94.3
yes	1	0.8	0.5	-0.2	0.1	94.9	95.3	95.0	0.2	-0.3	0.1	94.9	94.7	95.4	0.3	-0.4	0.3	94.5	94.4	94.5
yes	1	0.9	-0.2	-0.2	-0.2	94.9	94.5	95.1	-0.3	-0.4	-0.3	94.5	94.6	94.7	0.2	-0.6	-0.8	94.4	94.1	94.5
yes	1.5	0.9	-0.6	-0.5	-0.3	95.3	94.5	94.9	-1.1	-0.8	-0.4	95.3	94.4	94.6	-1.8	0.1	-0.5	94.3	94.6	94.5
yes	3	0.9	0.4	-0.2	-0.2	95.2	95.5	95.3	1.0	-0.1	-0.3	95.2	94.6	95.1	2.4	-0.1	0.0	94.7	93.9	94.3
no	0.1	0.9	-0.4	-0.1	-0.3	95.3	95.1	94.9	-0.4	-0.5	0.0	94.9	94.9	94.6	-0.7	-0.2	-0.7	95.4	94.1	94.3
no	1	0.2	0.0	0.3	0.0	95.0	95.3	94.6	-0.6	0.0	-0.4	94.2	95.1	94.6	0.3	0.1	-0.5	94.5	94.4	94.1
no	1	0.4	-0.4	-0.1	0.2	94.8	94.7	94.7	-0.5	0.1	-0.1	95.1	94.4	94.7	-0.7	0.0	0.4	94.3	94.7	94.7
no	1	0.6	0.4	0.2	0.1	94.7	95.1	95.0	0.1	0.5	0.0	94.9	95.0	94.7	-0.1	0.5	0.8	94.8	95.2	94.7
no	1	0.8	0.3	0.2	-0.1	94.7	94.6	95.2	-0.3	0.2	0.0	95.1	94.2	94.9	-0.3	-0.3	0.1	94.5	95.0	94.9
no	1	0.9	0.2	0.2	0.5	94.9	95.4	95.4	0.3	0.4	0.2	94.7	94.7	95.3	0.7	1.0	0.4	94.5	94.7	94.7
no	1.5	0.9	-0.5	0.2	0.2	95.0	95.2	94.8	-0.3	-0.2	0.3	94.6	95.3	94.7	0.4	-0.2	0.6	94.5	94.3	95.1
no	3	0.9	0.1	0.2	0.0	94.3	94.8	94.7	0.7	0.2	0.2	94.6	94.5	94.9	1.7	0.2	0.6	94.0	94.6	94.7

[a] Internal validation sample

[b] For varying sampling strategies of the internal validation sample, R: random, SR: stratified random, E: extremes

Validation regression calibration

The main results of validation regression calibration were shown in the main text. Web Figure 2 shows the mean squared error of the association between visceral adipose tissue and insulin resistance using an internal validation sample of 25% of the main study's sample size. Web Table 4 shows the mean squared error of the association under study in the scenarios where R-squared was equal to 0.9 or skewness was equal to 1.0, that were left out the main text for brevity. Web Table 5 shows the percentage bias and coverage of the association under study in the scenarios where R-squared was equal to 0.9 or skewness was equal to 1.0.

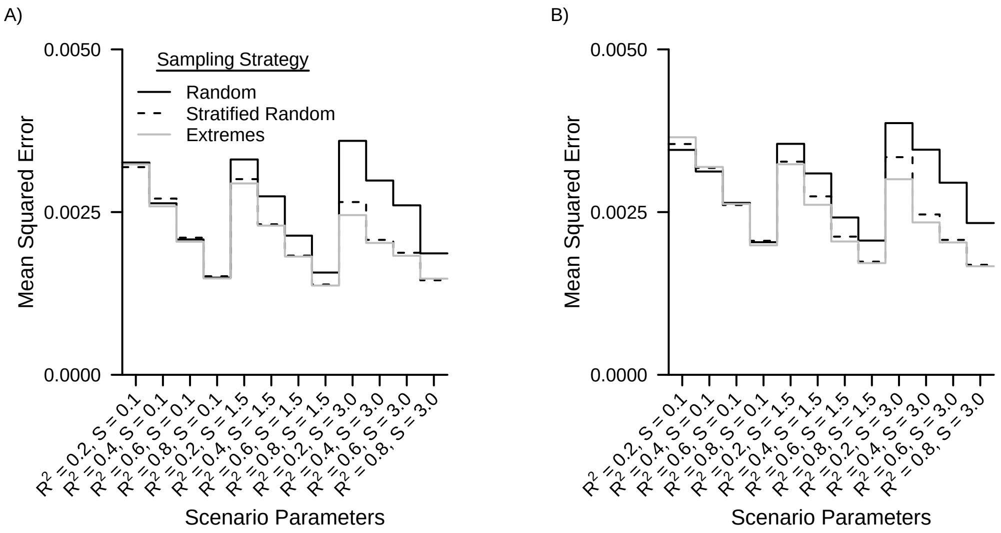

Web Figure 2. Nested loop plot of the mean squared errors in the analysis using validation regression calibration to correct for the measurement error for the three different sampling strategies. A) Linear measurement error model and an internal validation sample of 25% of the main study; and B) Non-linear measurement error model and an internal validation sample of 25% of the main study. Order from outer to inner loops: Skewness of the residual errors of the gold standard measure (S, 3 levels, increasing); R-squared of the measurement error model (R^2, 4 levels, increasing).

Web Table 4. Mean squared error of the estimated association between visceral adipose tissue and insulin resistance in the validation regression calibration analysis

Scenario			IVS[a] 40% of main study			IVS[a] 25% of main study			IVS[a] 10% of main study		
Linear	Skewness	R^2	Mean squared error[b]			Mean squared error[b]			Mean squared error[b]		
			R	*SR*	*E*	*R*	*SR*	*E*	*R*	*SR*	*E*
**yes**	**0.1**	**0.9**	0.0011	0.0011	0.0011	0.0012	0.0012	0.0012	0.0014	0.0013	0.0013
**yes**	**1**	**0.2**	0.0022	0.0021	0.0020	0.0033	0.0030	0.0029	0.0073	0.0058	0.0057
**yes**	**1**	**0.4**	0.0019	0.0018	0.0017	0.0025	0.0023	0.0023	0.0052	0.0038	0.0037
**yes**	**1**	**0.6**	0.0016	0.0014	0.0014	0.0021	0.0017	0.0017	0.0035	0.0025	0.0025
**yes**	**1**	**0.8**	0.0012	0.0012	0.0012	0.0014	0.0013	0.0013	0.0019	0.0016	0.0016
**yes**	**1**	**0.9**	0.0011	0.0011	0.0011	0.0012	0.0011	0.0011	0.0014	0.0012	0.0013
**yes**	**1.5**	**0.9**	0.0011	0.0010	0.0010	0.0012	0.0011	0.0011	0.0015	0.0013	0.0013
**yes**	**3**	**0.9**	0.0012	0.0011	0.0011	0.0014	0.0013	0.0013	0.0022	0.0016	0.0015
**no**	**0.1**	**0.9**	0.0014	0.0013	0.0013	0.0016	0.0015	0.0016	0.0021	0.0020	0.0024
**no**	**1**	**0.2**	0.0022	0.0021	0.0022	0.0034	0.0032	0.0032	0.0081	0.0070	0.0066
**no**	**1**	**0.4**	0.0021	0.0019	0.0019	0.0030	0.0027	0.0027	0.0067	0.0052	0.0047
**no**	**1**	**0.6**	0.0018	0.0016	0.0016	0.0025	0.0021	0.0021	0.0048	0.0036	0.0032
**no**	**1**	**0.8**	0.0015	0.0014	0.0014	0.0019	0.0016	0.0016	0.0031	0.0023	0.0023
**no**	**1**	**0.9**	0.0013	0.0012	0.0012	0.0015	0.0014	0.0014	0.0021	0.0017	0.0018
**no**	**1.5**	**0.9**	0.0013	0.0013	0.0012	0.0016	0.0014	0.0013	0.0024	0.0019	0.0017
**no**	**3**	**0.9**	0.0016	0.0015	0.0014	0.0020	0.0020	0.0016	0.0043	0.0031	0.0024

[a] Internal validation sample
[b] For varying sampling strategies of the internal validation sample, R: random, SR: stratified random, E: extremes

Web Table 5. Percentage bias and coverage of the estimated association between visceral adipose tissue and insulin resistance by application of validation regression calibration

Scenario			IVS[a]40% of main study						IVS[a]25% of main study						IVS[a]10% of main study					
Linear	Skewness	R^2	Percentage bias[b](%)			Coverage[b](%)			Percentage bias[b](%)			Coverage[b](%)			Percentage bias[b](%)			Coverage[b](%)		
			R	*SR*	*E*	*R*	*SR*	*E*	*R*	*SR*	*E*	*R*	*SR*	*E*	*R*	*SR*	*E*	*R*	*SR*	*E*
yes	0.1	0.9	-0.3	0.0	0.5	95.1	94.9	95.1	-0.5	-0.3	0.5	94.9	94.8	94.9	-0.7	-0.3	0.1	94.6	93.8	94.7
yes	1	0.2	0.3	-0.1	0.1	94.9	94.6	94.8	0.1	-0.4	0.0	95.2	94.2	94.2	0.9	-0.9	0.2	94.5	94.7	94.3
yes	1	0.4	-0.1	-0.6	-0.3	94.7	94.9	94.5	0.2	-0.7	-0.7	94.7	94.9	94.1	-0.6	-0.4	-0.3	94.7	94.5	94.3
yes	1	0.6	0.0	-0.2	-0.4	95.1	94.6	95.1	-0.2	-0.6	-0.2	94.6	94.4	95.3	0.8	-0.3	-0.3	94.8	94.7	94.3
yes	1	0.8	0.5	-0.2	0.1	94.9	95.3	95.0	0.2	-0.3	0.1	94.9	94.7	95.4	0.3	-0.4	0.3	94.5	94.4	94.5
yes	1	0.9	-0.2	-0.2	-0.2	94.9	94.5	95.1	-0.3	-0.4	-0.3	94.5	94.6	94.7	0.2	-0.6	-0.8	94.4	94.1	94.5
yes	1.5	0.9	-0.6	-0.5	-0.3	95.3	94.5	94.9	-1.1	-0.8	-0.4	95.3	94.4	94.6	-1.8	0.1	-0.5	94.3	94.6	94.5
yes	3	0.9	0.4	-0.2	-0.2	95.2	95.5	95.3	1.0	-0.1	-0.3	95.2	94.6	95.1	2.4	-0.1	0.0	94.7	93.9	94.3
no	0.1	0.9	-0.4	-0.1	-0.3	95.3	95.1	94.9	-0.4	-0.5	0.0	94.9	94.9	94.6	-0.7	-0.2	-0.7	95.4	94.1	94.3
no	1	0.2	0.0	0.3	0.0	95.0	95.3	94.6	-0.6	0.0	-0.4	94.2	95.1	94.6	0.3	0.1	-0.5	94.5	94.4	94.1
no	1	0.4	-0.4	-0.1	0.2	94.8	94.7	94.7	-0.5	0.1	-0.1	95.1	94.4	94.7	-0.7	0.0	0.4	94.3	94.7	94.7
no	1	0.6	0.4	0.2	0.1	94.7	95.1	95.0	0.1	0.5	0.0	94.9	95.0	94.7	-0.1	0.5	0.8	94.8	95.2	94.7
no	1	0.8	0.3	0.2	-0.1	94.7	94.6	95.2	-0.3	0.2	0.0	95.1	94.2	94.9	-0.3	-0.3	0.1	94.5	95.0	94.9
no	1	0.9	0.2	0.2	0.5	94.9	95.4	95.4	0.3	0.4	0.2	94.7	94.7	95.3	0.7	1.0	0.4	94.5	94.7	94.7
no	1.5	0.9	-0.5	0.2	0.2	95.0	95.2	94.8	-0.3	-0.2	0.3	94.6	95.3	94.7	0.4	-0.2	0.6	94.5	94.3	95.1
no	3	0.9	0.1	0.2	0.0	94.3	94.8	94.7	0.7	0.2	0.2	94.6	94.5	94.9	1.7	0.2	0.6	94.0	94.6	94.7

[a] Internal validation sample

[b] For varying sampling strategies of the internal validation sample, R: random, SR: stratified random, E: extremes

Efficient regression calibration

The results of the application of efficient regression calibration for measurement error correction were as follows. Web Figure 3 shows the mean squared error of the association between visceral adipose tissue and insulin resistance using an internal validation sample of 10%, or 40% of the main study's sample size. Web Figure 4 shows the mean squared error of the association between visceral adipose tissue and insulin resistance using an internal validation sample of 25% of the main study's sample size. Web Table 6 shows the mean squared error of the association under study in the scenarios where R-squared was equal to 0.9 or skewness was equal to 1.0, that were left out Web Figure 3 and 4 for comparability with Figure 5-6 in the main text. Web Table 7 shows the percentage bias and coverage of the association between visceral adipose tissue and insulin resistance using an internal validation sample of 10%, 25% or 40% of the main study's sample size.

Web Table 6. Mean squared error of the estimated association between visceral adipose tissue and insulin resistance in the efficient regression calibration analysis

Scenario			IVS[a] 40% of main study			IVS[a] 25% of main study			IVS[a] 10% of main study		
**Linear**	**Skewness**	R^2	Mean squared error[b]			Mean squared error[b]			Mean squared error[b]		
			R	*SR*	*E*	*R*	*SR*	*E*	*R*	*SR*	*E*
**yes**	**0.1**	**0.9**	0.0012	0.0012	0.0012	0.0013	0.0013	0.0013	0.0014	0.0014	0.0014
**yes**	**1**	**0.2**	0.0023	0.0022	0.0022	0.0033	0.0032	0.0032	0.0072	0.0059	0.0060
**yes**	**1**	**0.4**	0.0020	0.0019	0.0019	0.0026	0.0025	0.0025	0.0049	0.0038	0.0038
**yes**	**1**	**0.6**	0.0018	0.0016	0.0016	0.0022	0.0019	0.0019	0.0033	0.0026	0.0026
**yes**	**1**	**0.8**	0.0014	0.0013	0.0012	0.0015	0.0014	0.0014	0.0020	0.0017	0.0016
**yes**	**1**	**0.9**	0.0012	0.0012	0.0012	0.0013	0.0012	0.0012	0.0015	0.0013	0.0014
**yes**	**1.5**	**0.9**	0.0012	0.0011	0.0011	0.0013	0.0012	0.0012	0.0015	0.0013	0.0013
**yes**	**3**	**0.9**	0.0013	0.0011	0.0011	0.0014	0.0012	0.0012	0.0021	0.0014	0.0013
**no**	**0.1**	**0.9**	0.0015	0.0014	0.0014	0.0017	0.0017	0.0017	0.0022	0.0021	0.0024
**no**	**1**	**0.2**	0.0023	0.0022	0.0022	0.0035	0.0033	0.0033	0.0082	0.0073	0.0069
**no**	**1**	**0.4**	0.0022	0.0021	0.0020	0.0031	0.0029	0.0029	0.0064	0.0053	0.0050
**no**	**1**	**0.6**	0.0019	0.0018	0.0018	0.0025	0.0023	0.0023	0.0043	0.0037	0.0034
**no**	**1**	**0.8**	0.0017	0.0015	0.0015	0.0020	0.0018	0.0018	0.0030	0.0024	0.0024
**no**	**1**	**0.9**	0.0014	0.0013	0.0013	0.0017	0.0015	0.0015	0.0022	0.0018	0.0019
**no**	**1.5**	**0.9**	0.0015	0.0013	0.0013	0.0017	0.0014	0.0014	0.0023	0.0019	0.0017
**no**	**3**	**0.9**	0.0016	0.0014	0.0013	0.0000	0.0016	0.0015	0.0037	0.0025	0.0020

[a] Internal validation sample
[b] For varying sampling strategies of the internal validation sample, R: random, SR: stratified random, E: extremes

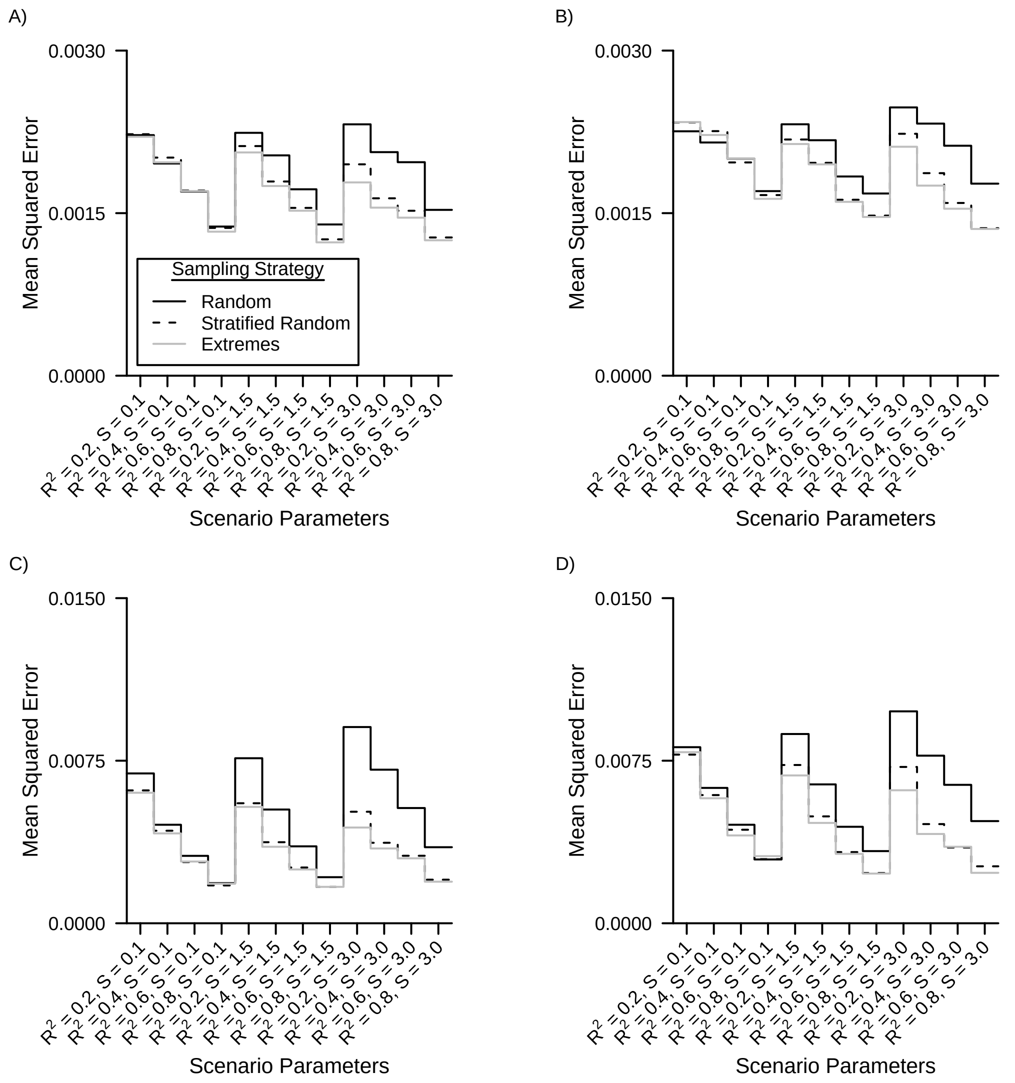

Web Figure 3. Nested loop plot of the mean squared errors in the analysis using efficient regression calibration to correct for the measurement error for the three different sampling strategies. A) Linear measurement error model and an internal validation sample of 40% of the main study; B) Non-linear measurement error model and an internal validation sample of 40% of the main study; C) Linear measurement error model and an internal validation sample of 10% of the main study; and D) Non-linear measurement error model and an internal validation sample of 10% of the main study. Order from outer to inner loops: Skewness of the residual errors of the gold standard measure (S, 3 levels, increasing); R-squared of the measurement error model (R^2, 4 levels, increasing).

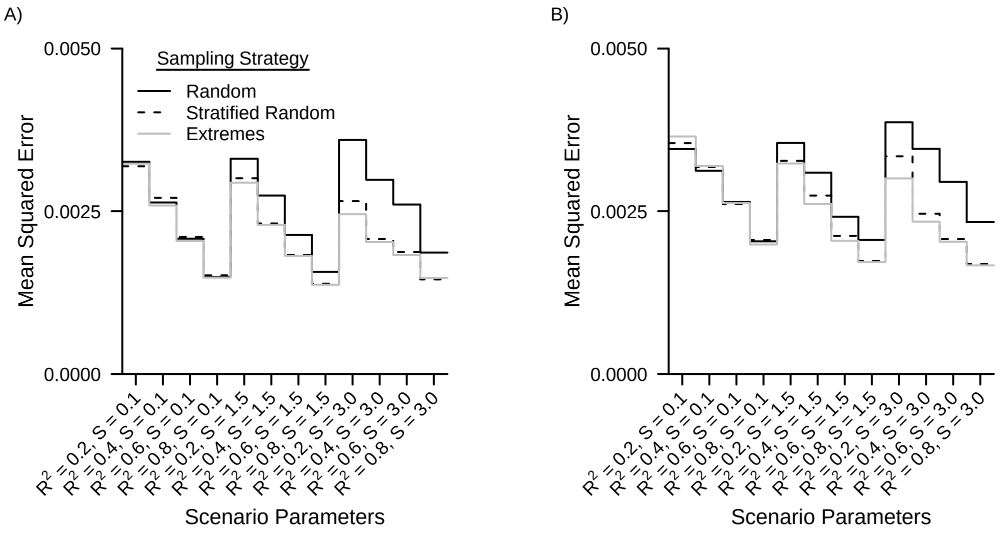

Web Figure 4. Nested loop plot of the mean squared errors in the analysis using efficient regression calibration to correct for the measurement error for the three different sampling strategies. A) Linear measurement error model and an internal validation sample of 25% of the main study; and B) Non-linear measurement error model and an internal validation sample of 25% of the main study. Order from outer to inner loops: Skewness of the residual errors of the gold standard measure (S, 3 levels, increasing); R-squared of the measurement error model (R^2, 4 levels, increasing).

Web Table 7. Percentage bias and coverage of the estimated association between visceral adipose tissue and insulin resistance by application of efficient regression calibration

Scenario			IVS[a]40% of main study						IVS[a]25% of main study						IVS[a]10% of main study					
**Linear**	**Skewness**	R^2	Percentage bias[b](%)			Coverage[b](%)			Percentage bias[b](%)			Coverage[b](%)			Percentage bias[b](%)			Coverage[b](%)		
			R	*SR*	*E*	*R*	*SR*	*E*	*R*	*SR*	*E*	*R*	*SR*	*E*	*R*	*SR*	*E*	*R*	*SR*	*E*
**yes**	**0.1**	**0.2**	-1.2	-0.3	-0.4	93.4	92.7	92.6	-2.2	-0.6	-0.7	93.9	93.0	92.4	-6.3	-2.5	-2.3	92.9	93.2	92.2
**yes**	**0.1**	**0.4**	-0.6	0.0	-0.1	92.1	91.2	90.3	-0.9	-0.2	-0.3	93.4	90.8	91.1	-1.9	0.0	0.0	93.6	92.0	91.5
**yes**	**0.1**	**0.6**	0.6	0.8	0.5	90.9	89.7	88.7	0.6	0.9	0.7	92.7	90.4	89.6	1.4	1.3	1.2	93.8	92.3	91.5
**yes**	**0.1**	**0.8**	0.0	0.1	0.2	89.6	88.8	88.5	0.0	0.1	0.4	91.9	89.5	89.6	0.8	0.3	0.7	94.2	92.6	91.5
**yes**	**0.1**	**0.9**	-0.1	0.0	0.1	88.5	87.1	87.0	-0.1	-0.1	0.2	90.6	89.0	88.1	0.2	0.2	0.3	93.2	91.4	91.1
**yes**	**1**	**0.2**	-0.6	-0.8	-0.5	93.5	92.3	91.9	-1.9	-1.7	-1.1	93.5	92.1	91.2	-5.8	-4.5	-3.1	92.3	91.9	91.1
**yes**	**1**	**0.4**	-0.5	-1.5	-1.2	92.0	90.2	90.0	-0.6	-2.4	-2.2	92.9	90.7	89.7	-1.7	-4.5	-4.2	92.3	91.5	90.2
**yes**	**1**	**0.6**	-0.2	-1.6	-1.9	91.0	89.0	88.5	-0.3	-3.0	-2.7	91.6	89.7	89.4	0.7	-5.3	-5.6	93.4	89.6	88.7
**yes**	**1**	**0.8**	0.1	-1.6	-1.6	90.5	88.8	88.7	0.1	-2.6	-2.2	91.7	89.6	90.2	1.0	-4.7	-3.9	93.5	90.4	90.2
**yes**	**1**	**0.9**	-0.1	-1.1	-1.1	88.7	87.5	86.7	0.0	-1.7	-1.4	90.4	88.8	88.5	0.6	-3.1	-2.3	92.7	90.2	90.1
**yes**	**1.5**	**0.2**	-1.1	-1.1	-0.7	93.7	92.7	92.5	-2.2	-2.0	-1.7	93.4	92.5	92.0	-5.8	-5.6	-4.7	91.9	92.5	91.4
**yes**	**1.5**	**0.4**	-0.2	-1.5	-1.6	91.9	91.0	91.0	-0.4	-3.1	-2.7	92.4	90.9	90.4	-1.3	-7.4	-7.4	92.3	88.9	88.8
**yes**	**1.5**	**0.6**	0.3	-2.4	-2.4	90.8	89.4	88.2	0.7	-4.5	-3.9	92.1	88.9	88.4	2.1	-8.9	-8.9	92.7	86.5	85.7
**yes**	**1.5**	**0.8**	0.3	-2.4	-2.9	89.3	87.7	87.1	0.4	-4.3	-3.9	91.7	88.2	87.6	1.9	-7.9	-7.0	92.7	87.3	86.8
**yes**	**1.5**	**0.9**	-0.2	-2.2	-2.3	89.4	86.7	86.6	-0.2	-3.4	-3.1	90.6	87.9	87.6	0.6	-5.5	-4.4	92.5	89.0	88.5
**yes**	**3**	**0.2**	-1.0	-1.4	-1.5	93.0	92.1	92.5	-2.1	-3.2	-2.6	93.0	92.2	92.4	-5.4	-9.5	-8.0	90.7	88.5	88.8
**yes**	**3**	**0.4**	0.2	-3.3	-3.7	92.0	90.9	89.7	0.4	-7.1	-5.9	92.3	88.4	88.3	0.8	-16.2	-16.0	90.9	77.8	79.0
**yes**	**3**	**0.6**	0.7	-5.3	-6.5	89.8	86.3	85.3	1.5	-9.9	-9.7	90.2	83.0	83.5	5.3	-19.2	-19.2	90.0	69.2	70.2
**yes**	**3**	**0.8**	0.9	-5.1	-6.4	89.2	85.2	83.4	1.8	-8.4	-8.9	90.2	83.1	81.9	6.2	-14.3	-13.3	90.2	76.2	77.4
**yes**	**3**	**0.9**	0.6	-3.6	-4.4	89.1	85.8	84.8	1.3	-5.5	-5.7	89.8	85.3	84.9	4.0	-8.9	-7.5	91.4	83.5	84.6
**no**	**0.1**	**0.2**	-0.9	-0.4	-0.3	94.2	93.7	93.4	-2.0	-1.2	-0.7	93.6	93.3	92.7	-7.9	-4.8	-2.8	93.0	92.7	92.9
**no**	**0.1**	**0.4**	-0.3	-0.3	-0.7	93.6	91.8	91.4	-0.9	-0.6	-1.4	93.0	92.2	91.6	-4.2	-1.9	-3.4	93.2	92.7	90.8
**no**	**0.1**	**0.6**	-0.3	-0.7	-1.2	92.2	91.3	90.0	-0.4	-0.4	-2.5	92.5	91.6	89.8	-2.2	0.3	-3.0	93.2	92.5	90.7
**no**	**0.1**	**0.8**	0.2	-0.1	0.3	91.3	89.4	89.6	0.4	0.6	1.0	92.4	90.5	90.3	0.7	2.7	4.9	93.8	92.6	92.2
**no**	**0.1**	**0.9**	-0.2	-1.5	-0.1	90.4	89.1	88.7	-0.2	-1.3	1.3	91.3	89.6	89.1	0.3	-0.3	4.5	93.7	92.2	92.3
**no**	**1**	**0.2**	-1.1	-0.4	-0.6	94.3	93.8	92.8	-2.8	-1.4	-1.6	93.6	93.4	93.1	-6.9	-4.2	-4.5	92	93.2	92.6
**no**	**1**	**0.4**	-1.4	-1.2	-1.2	93.3	92.2	91.7	-2.3	-1.9	-2.9	93.7	91.9	91.1	-5.4	-4.1	-6.8	92.1	91.8	89.9
**no**	**1**	**0.6**	0.2	-0.9	-1.7	92.1	90.7	89.9	-0.1	-1.4	-3.8	92.9	91.1	89.3	-0.7	-1.9	-5.9	93.3	91.5	89.5
**no**	**1**	**0.8**	0.3	-2.2	-2.1	90.3	88.5	88.0	0.3	-2.6	-2.5	91.8	89.4	89.2	1.0	-3.0	-0.9	93.0	91.0	90.8
**no**	**1**	**0.9**	0.3	-3.4	-1.4	90.0	88.1	87.7	0.5	-4.1	-1.2	91.3	88.4	89.1	1.4	-5.4	-0.5	93.2	90.1	91.0
**no**	**1.5**	**0.2**	-1.3	-0.6	-0.9	94.1	93.5	93.6	-2.3	-1.6	-1.6	94.3	93.9	93.3	-7.3	-4.8	-4.5	92.4	92.9	92.3
**no**	**1.5**	**0.4**	-1.4	-1.5	-1.8	93.1	91.9	91.5	-2.1	-2.5	-3.5	93.0	91.7	91.2	-5.5	-5.0	-9.0	92.5	91.1	88.8
**no**	**1.5**	**0.6**	-0.1	-1.9	-2.6	92.7	91.4	90.6	-0.2	-3.2	-5.5	93.5	90.9	89.1	-0.4	-5.5	-10.0	93.3	91.1	87.3
**no**	**1.5**	**0.8**	-0.1	-4.0	-3.7	90.6	87.3	87.0	0.2	-5.3	-5.1	91.4	88.1	87.2	1.5	-7.3	-5.6	91.9	88.9	88.2
**no**	**1.5**	**0.9**	0.0	-4.9	-3.1	89.7	86.5	86.9	0.4	-6.5	-3.4	90.8	87.0	87.7	1.9	-9.5	-4.3	92.8	85.5	88.9
**no**	**3**	**0.2**	-1.3	-0.8	-0.7	93.5	92.7	92.8	-2.7	-1.6	-1.5	93.7	93.0	92.9	-8.1	-4.6	-5.6	91.8	92.1	92.2
**no**	**3**	**0.4**	-0.9	-2.5	-2.7	92.6	91.1	90.9	-1.7	-4.4	-5.0	92.5	91.0	89.8	-4.2	-9.5	-12.8	91.1	88.0	85.1
**no**	**3**	**0.6**	-0.2	-4.4	-4.8	91.4	89.3	88.8	-0.1	-7.5	-9.3	91.7	87.4	85.3	1.1	-13.3	-18.0	90.5	82.1	76.0
**no**	**3**	**0.8**	0.8	-6.6	-6.4	90.1	85.8	86.0	1.4	-9.8	-9.5	90.8	83.5	83.2	5.3	-15.8	-13.7	90.2	76.1	79.0
**no**	**3**	**0.9**	0.6	-8.0	-6.8	88.6	82.1	83.5	1.6	-11.3	-8.6	89.6	78.8	82.1	5.6	-17.6	-12.8	89.6	69.9	78.9

[a] Internal validation sample

[b] For varying sampling strategies of the internal validation sample, R: random, SR: stratified random, E: extremes

Standard regression calibration

The results of the application of standard regression calibration for measurement error correction were as follows. Web Table 8 shows the mean squared error of the association between visceral adipose tissue and insulin resistance using an internal validation sample of 10%, 25% or 40% of the main study's sample size. Web Table 9 shows the percentage bias and coverage of the association between visceral adipose tissue and insulin resistance using an internal validation sample of 10%, 25% or 40% of the main study's sample size.

Web Table 8. Mean squared error of the estimated association between visceral adipose tissue and insulin resistance in the standard regression calibration analysis

Scenario			IVS[a]40% of main study			IVS[a]25% of main study			IVS[a]10% of main study		
Linear	Skewness	R^2	Mean squared error[b]			Mean squared error[b]			Mean squared error[b]		
			R	*SR*	*E*	*R*	*SR*	*E*	*R*	*SR*	*E*
**yes**	**0.1**	**0.2**	0.013	0.011	0.011	0.020	0.012	0.011	2.144	0.042	0.015
**yes**	**0.1**	**0.4**	0.005	0.005	0.005	0.006	0.005	0.005	0.013	0.007	0.006
**yes**	**0.1**	**0.6**	0.003	0.003	0.003	0.003	0.003	0.003	0.004	0.003	0.003
**yes**	**0.1**	**0.8**	0.002	0.002	0.002	0.002	0.002	0.002	0.002	0.002	0.002
**yes**	**0.1**	**0.9**	0.001	0.001	0.001	0.001	0.001	0.001	0.001	0.001	0.001
**yes**	**1**	**0.2**	0.014	0.011	0.011	0.094	0.012	0.011	5.839	0.107	0.016
**yes**	**1**	**0.4**	0.005	0.005	0.004	0.006	0.005	0.005	0.013	0.006	0.006
**yes**	**1**	**0.6**	0.003	0.003	0.002	0.003	0.003	0.003	0.005	0.003	0.003
**yes**	**1**	**0.8**	0.002	0.001	0.001	0.002	0.002	0.001	0.002	0.002	0.002
**yes**	**1**	**0.9**	0.001	0.001	0.001	0.001	0.001	0.001	0.001	0.001	0.001
**yes**	**1.5**	**0.2**	0.013	0.011	0.011	0.019	0.012	0.011	2.667	0.067	0.013
**yes**	**1.5**	**0.4**	0.005	0.004	0.004	0.006	0.004	0.004	0.641	0.006	0.005
**yes**	**1.5**	**0.6**	0.003	0.002	0.002	0.003	0.003	0.002	0.006	0.003	0.003
**yes**	**1.5**	**0.8**	0.002	0.001	0.001	0.002	0.002	0.002	0.002	0.002	0.002
**yes**	**1.5**	**0.9**	0.001	0.001	0.001	0.001	0.001	0.001	0.002	0.001	0.001
**yes**	**3**	**0.2**	0.017	0.011	0.010	0.030	0.011	0.011	20.259	1.602	0.016
**yes**	**3**	**0.4**	0.007	0.005	0.004	0.011	0.005	0.005	11.778	0.007	0.006
**yes**	**3**	**0.6**	0.004	0.003	0.003	0.005	0.003	0.003	0.012	0.005	0.005
**yes**	**3**	**0.8**	0.002	0.002	0.002	0.002	0.002	0.002	0.005	0.003	0.003
**yes**	**3**	**0.9**	0.001	0.001	0.001	0.001	0.001	0.001	0.002	0.002	0.002
**no**	**0.1**	**0.2**	0.167	0.025	0.023	0.355	0.030	0.025	6.808	0.247	0.045
**no**	**0.1**	**0.4**	0.010	0.009	0.008	0.012	0.009	0.009	0.143	0.014	0.010
**no**	**0.1**	**0.6**	0.005	0.005	0.004	0.005	0.005	0.004	0.011	0.007	0.005
**no**	**0.1**	**0.8**	0.003	0.002	0.002	0.003	0.003	0.003	0.004	0.003	0.004
**no**	**0.1**	**0.9**	0.002	0.002	0.002	0.002	0.002	0.002	0.002	0.002	0.003
**no**	**1**	**0.2**	0.036	0.020	0.019	46.762	0.023	0.021	19.508	8.612	1.576
**no**	**1**	**0.4**	0.009	0.008	0.007	0.012	0.008	0.008	1.074	0.012	0.008
**no**	**1**	**0.6**	0.004	0.004	0.004	0.005	0.004	0.004	0.014	0.005	0.004
**no**	**1**	**0.8**	0.002	0.002	0.002	0.003	0.002	0.002	0.004	0.003	0.003
**no**	**1**	**0.9**	0.002	0.002	0.002	0.002	0.002	0.002	0.002	0.002	0.002
**no**	**1.5**	**0.2**	0.244	0.021	0.020	1.050	0.023	0.021	106.246	9.679	0.116
**no**	**1.5**	**0.4**	0.009	0.008	0.007	0.011	0.008	0.007	10.060	0.012	0.008
**no**	**1.5**	**0.6**	0.004	0.004	0.004	0.005	0.004	0.004	0.012	0.005	0.004
**no**	**1.5**	**0.8**	0.003	0.002	0.002	0.003	0.002	0.002	0.005	0.003	0.003
**no**	**1.5**	**0.9**	0.002	0.002	0.002	0.002	0.002	0.002	0.003	0.002	0.002
**no**	**3**	**0.2**	0.036	0.022	0.020	0.402	0.029	0.022	66.44	0.601	0.085
**no**	**3**	**0.4**	0.011	0.007	0.007	0.019	0.008	0.007	2.702	0.021	0.008
**no**	**3**	**0.6**	0.006	0.004	0.004	0.007	0.004	0.005	0.082	0.006	0.006
**no**	**3**	**0.8**	0.003	0.003	0.003	0.004	0.003	0.003	0.012	0.004	0.003
**no**	**3**	**0.9**	0.002	0.002	0.002	0.002	0.003	0.002	0.005	0.004	0.003

[a] Internal validation sample
[b] For varying sampling strategies of the internal validation sample, R: random, SR: stratified random, E: extremes

Web Table 9. Percentage bias and coverage of the estimated association between visceral adipose tissue and insulin resistance by application of standard regression calibration

Scenario			IVS[a]40% of main study						IVS[a]25% of main study						IVS[a]10% of main study					
**Linear**	**Skewness**	R^2	Percentage bias[b](%)			Coverage[b](%)			Percentage bias[b](%)			Coverage[b](%)			Percentage bias[b](%)			Coverage[b](%)		
			R	*SR*	*E*	*R*	*SR*	*E*	*R*	*SR*	*E*	*R*	*SR*	*E*	*R*	*SR*	*E*	*R*	*SR*	*E*
**yes**	**0.1**	**0.2**	3.5	1.2	0.9	96.9	96.9	96.8	7.4	2.1	1.6	96.8	97.0	97.0	26.9	7.3	4.4	94.9	96.7	97
**yes**	**0.1**	**0.4**	0.7	0.4	0.2	95.9	95.9	95.8	1.8	0.8	0.7	96.4	95.9	95.9	6.9	3.4	2.5	95.6	96.0	95.7
**yes**	**0.1**	**0.6**	1.5	1.4	1.3	95.9	96.0	96.1	2.0	1.8	1.6	95.9	96.0	96.2	4.3	2.8	2.6	96.3	96.3	96.7
**yes**	**0.1**	**0.8**	0.3	0.2	0.3	95.8	95.5	95.7	0.4	0.3	0.5	95.6	95.6	95.9	1.2	0.6	1.1	95.9	95.6	95.8
**yes**	**0.1**	**0.9**	0.1	0.0	0.0	94.7	94.7	94.9	0.1	0.1	0.1	94.9	95.0	95.2	0.4	0.3	0.3	95.3	94.9	95.2
**yes**	**1**	**0.2**	3.4	-0.2	-0.7	96.5	96.4	96.3	9.1	-0.1	0.1	96.5	96.3	96.5	10.6	2.9	1.9	94.5	95.6	95.9
**yes**	**1**	**0.4**	1.0	-2.2	-2.4	95.9	95.8	96.1	2.3	-3.2	-2.5	96.2	95.3	95.7	9.2	-3.5	-3.6	94.7	94.2	94.1
**yes**	**1**	**0.6**	0.1	-3.3	-3.6	95.7	95.3	95.4	0.7	-4.8	-4.3	95.3	94.7	95.1	3.5	-6.7	-7.1	95.3	92.2	93.0
**yes**	**1**	**0.8**	-0.1	-2.7	-3.0	95.7	95.5	95.6	0.2	-4.0	-3.5	95.8	95.0	95.4	1.5	-5.8	-5.2	95.7	93.8	94.3
**yes**	**1**	**0.9**	0.0	-1.7	-1.8	95.2	95.0	95.0	0.1	-2.5	-2.1	94.9	94.8	94.9	0.7	-3.7	-2.9	94.8	94.6	94.9
**yes**	**1.5**	**0.2**	2.2	-2.2	-2.3	96.9	96.7	96.9	6.7	-2.6	-1.9	96.3	96.4	97.0	30.8	1.2	-2.5	94.0	94.6	95.5
**yes**	**1.5**	**0.4**	1.7	-4.4	-4.5	96.2	95.8	96.2	3.5	-6.8	-5.3	96.2	95.1	95.7	20.5	-9.6	-9.8	94.6	90.5	92.1
**yes**	**1.5**	**0.6**	1.4	-4.9	-5.5	96.1	95.2	95.1	2.3	-7.8	-6.9	95.6	93.5	94.3	6.8	-12.0	-12.3	94.7	88.5	89.7
**yes**	**1.5**	**0.8**	0.5	-4.4	-5.0	95.9	95.0	94.9	1.1	-6.7	-6.1	95.8	93.7	93.9	2.9	-10.2	-9.3	95.6	90.2	90.8
**yes**	**1.5**	**0.9**	-0.1	-3.4	-3.7	95.5	95.1	95.2	0.1	-4.8	-4.3	95.2	94.8	95.0	1.0	-7.0	-5.8	95.4	93.0	93.5
**yes**	**3**	**0.2**	6.4	-5.6	-6.0	96.4	95.9	96.6	14.1	-9.2	-5.3	95.8	94.7	96.2	108.3	-22.6	-12.2	92.5	88.3	91.7
**yes**	**3**	**0.4**	4.1	-9.8	-11.4	95.2	93.5	93.8	8.6	-16.4	-13.2	95.0	89.6	92.3	51.9	-25.6	-25.6	92.4	74.3	77.7
**yes**	**3**	**0.6**	2.6	-11.6	-14.2	94.3	91.3	90.0	5.2	-18.1	-17.3	93.4	84.2	86.3	15.4	-27.5	-27.7	92.0	64.6	66.8
**yes**	**3**	**0.8**	1.6	-9.5	-11.7	94.4	91.8	90.0	2.8	-13.9	-14.0	93.2	86.7	87.1	8.6	-20.0	-19.0	91.3	74.8	78.1
**yes**	**3**	**0.9**	0.7	-6.1	-7.5	94.6	93.2	92.5	1.4	-8.7	-8.8	94.2	91.0	90.9	4.2	-12.1	-10.9	93.4	86.9	88.7
**no**	**0.1**	**0.2**	5.6	1.9	0.0	97.3	97.2	97.2	16.4	4.1	1.3	96.9	97.3	97.3	45.8	16.7	7.1	94.7	96.6	97.0
**no**	**0.1**	**0.4**	3.0	1.2	-0.4	97.2	96.9	96.6	5.3	2.1	-0.9	97.4	97.2	96.9	16.4	6.7	-0.4	95.6	97.1	96.5
**no**	**0.1**	**0.6**	0.3	-0.2	-2.1	96.7	96.3	96.1	1.3	1.0	-3.3	96.6	96.3	96.0	6.5	5.2	-1.1	95.4	96.8	96.3
**no**	**0.1**	**0.8**	0.9	0.3	0.5	95.8	95.9	96.1	1.5	1.9	2.2	95.9	96.1	96.1	3.2	5.2	8.9	96.4	96.4	96.6
**no**	**0.1**	**0.9**	0.1	-2.0	0.4	95.6	95.1	95.5	0.2	-1.2	2.9	95.9	95.1	95.5	1.1	0.3	7.3	96.1	96.0	96.3
**no**	**1**	**0.2**	7.9	0.7	-0.3	97.4	97.3	97.2	-29.6	2.3	0.6	97.0	97.3	97.1	2.3	-5.5	-4.9	94.8	96.7	96.7
**no**	**1**	**0.4**	1.0	-2.8	-4.2	96.4	96.3	96.1	3.4	-2.9	-5.8	96.5	96.2	95.7	21.2	0.7	-7.9	94.3	95.4	94.3
**no**	**1**	**0.6**	1.9	-2.4	-3.9	96.5	96.2	96.0	2.9	-2.8	-6.8	96.5	95.9	95.0	9.0	-0.6	-7.3	95.7	95.4	94.1
**no**	**1**	**0.8**	0.8	-4.7	-4.0	95.7	94.8	95.1	1.5	-4.5	-4.0	95.5	95.2	94.9	3.7	-3.2	-0.2	95.3	94.4	95.5
**no**	**1**	**0.9**	0.6	-6.0	-2.7	95.6	94.1	95.1	0.8	-6.3	-1.5	95.7	94.2	95.5	2.1	-6.9	-0.5	95.9	92.9	95.5
**no**	**1.5**	**0.2**	9.8	0.3	-0.5	97.4	97.3	97.5	14.3	1.9	0.4	96.4	97.0	97.3	-79.8	39.5	4.5	94.4	95.9	96.4
**no**	**1.5**	**0.4**	1.0	-4.4	-5.4	96.5	96.2	96.2	3.7	-5.1	-7.8	96.0	95.9	95.5	-16.0	-3.0	-11.3	94.1	94.0	92.9
**no**	**1.5**	**0.6**	1.2	-5.7	-7.2	96.4	95.7	95.4	2.4	-7.1	-10.9	96.5	95.3	94.2	9.5	-6.9	-13.5	95.0	93.9	91.5
**no**	**1.5**	**0.8**	0.2	-8.3	-7.5	94.9	93.6	93.7	1.2	-9.1	-8.5	94.9	92.7	93.4	4.4	-9.5	-7.3	94.2	91.1	93.2
**no**	**1.5**	**0.9**	0.5	-8.8	-5.6	95.2	92.5	94.6	0.9	-10.0	-5.3	95.1	91.5	94.5	2.9	-11.9	-6.1	94.8	88.4	93.1
**no**	**3**	**0.2**	8.9	-1.5	-2.9	97.2	97.2	97.1	26.9	-0.5	-2.9	96.6	96.6	97.0	115.6	1.6	-1.5	93.9	94.9	95.4
**no**	**3**	**0.4**	3.3	-9.1	-9.2	96.5	95.1	95.7	8.8	-12.6	-13.4	95.9	93.6	94.1	29.2	-13.6	-22.0	92.9	88.9	86.1
**no**	**3**	**0.6**	2.3	-13.1	-13.9	95.2	92.9	92.5	5.1	-17.6	-20.0	94.5	88.9	87.4	20.1	-21.1	-28.0	92.8	80.5	75.1
**no**	**3**	**0.8**	1.9	-14.8	-14.1	94.6	89.2	90.1	3.7	-18.6	-17.5	93.6	84.1	85.9	12.3	-22.8	-21.2	91.6	73.5	79.5
**no**	**3**	**0.9**	1.0	-14.9	-12.7	94.1	86.1	89.3	2.3	-18.5	-14.1	93.3	80.0	87.1	7.6	-23.7	-19.0	90.4	66.1	78.7

[a] Internal validation sample
[b] For varying sampling strategies of the internal validation sample, R: random, SR: stratified random, E: extremes

References

[1] RJ Carroll, D Ruppert, LA Stefanski, et al. *Measurement error in nonlinear models: A modern perspective*. Chapman & Hall/CRC, Boca Raton, FL, 2nd edition, 2006.

[2] RH Keogh, PA Shaw, P Gustafson, et al. STRATOS guidance document on measurement error and misclassification of variables in observational epidemiology: Part 1—Basic theory and simple methods of adjustment. *Stat Med*, 39(16):2197–2231, 2020.

[3] RH Keogh and IR White. A toolkit for measurement error correction, with a focus on nutritional epidemiology. *Stat Med*, 33:2137–2155, 2014.

[4] B Rosner, D Spiegelman, and WC Willett. Correction of logistic regression relative risk estimates and confidence intervals for measurement error: the case of multiple covariates measured with error. *Am J Epidemiol*, 132(4):734–745, 1990.

[5] D Spiegelman, RJ Carroll, and V Kipnis. Efficient regression calibration for logistic regression in main study/internal validation study designs with an imperfect reference instrument. *Stat Med*, 20:139–160, 2001.

[6] L Nab. LindaNab/me_neo v1.0.4: Final code and output of the simulation study 'Sampling strategies for internal validation samples for exposure measurement error correction'. https://doi.org/10.5281/zenodo.4648989, March 2021.
